# Molecular simulations and Markov state modeling reveal the structural diversity and dynamics of a theophylline-binding RNA aptamer in its unbound state

**DOI:** 10.1371/journal.pone.0176229

**Published:** 2017-04-24

**Authors:** Becka M. Warfield, Peter C. Anderson

**Affiliations:** Department of Physical Sciences, University of Washington, Bothell, Washington, United States of America; Hong Kong University of Science and Technology, HONG KONG

## Abstract

RNA aptamers are oligonucleotides that bind with high specificity and affinity to target ligands. In the absence of bound ligand, secondary structures of RNA aptamers are generally stable, but single-stranded and loop regions, including ligand binding sites, lack defined structures and exist as ensembles of conformations. For example, the well-characterized theophylline-binding aptamer forms a highly stable binding site when bound to theophylline, but the binding site is unstable and disordered when theophylline is absent. Experimental methods have not revealed at atomic resolution the conformations that the theophylline aptamer explores in its unbound state. Consequently, in the present study we applied 21 microseconds of molecular dynamics simulations to structurally characterize the ensemble of conformations that the aptamer adopts in the absence of theophylline. Moreover, we apply Markov state modeling to predict the kinetics of transitions between unbound conformational states. Our simulation results agree with experimental observations that the theophylline binding site is found in many distinct binding-incompetent states and show that these states lack a binding pocket that can accommodate theophylline. The binding-incompetent states interconvert with binding-competent states through structural rearrangement of the binding site on the nanosecond to microsecond timescale. Moreover, we have simulated the complete theophylline binding pathway. Our binding simulations supplement prior experimental observations of slow theophylline binding kinetics by showing that the binding site must undergo a large conformational rearrangement after the aptamer and theophylline form an initial complex, most notably, a major rearrangement of the C27 base from a buried to solvent-exposed orientation. Theophylline appears to bind by a combination of conformational selection and induced fit mechanisms. Finally, our modeling indicates that when Mg^2+^ ions are present the population of binding-competent aptamer states increases more than twofold. This population change, rather than direct interactions between Mg^2+^ and theophylline, accounts for altered theophylline binding kinetics.

## Introduction

Many RNAs exist as large ensembles of conformations in solution when they are unbound[1–6]. Whereas in most proteins individual *α*-helices and *β*-sheets are unstable individually[7], individual helical secondary structural elements in RNA are often highly stable[8]. This stability enables RNAs to form partially folded states containing defined secondary structure but limited or no tertiary structure[4,8–10]. These partially folded states are often structurally heterogeneous, forming a complex structural landscape, and RNA molecules transition frequently between different conformational states in the landscape[11,12]. Such conformational transitions underlie several fundamental biological processes involving RNA[11,12], including metabolite sensing and gene regulation by riboswitches, RNA catalysis by ribozymes, and co-transcriptional RNA folding. For example, several ribozymes have been shown to exist as a distribution of conformational states, and only a minor fraction of the equilibrium population is in the catalytically active state; a conformational change is thus necessary for the active state to become populated[13–16].

Another class of RNA molecules that often occur as heterogeneous ensembles of conformations in the unbound state are RNA aptamers. Aptamers are single-stranded oligonucleotides whose function is to recognize and bind target molecules with high affinity and selectivity[17–22]. These targets range in size from small molecules to proteins and cells. When they are bound to their targets, aptamers form complex three-dimensional structures featuring intricate motifs and stable target binding sites[23,24]. The complexity of these structures enables aptamers to bind selectively to target molecules with dissociation constants as low as picomolar values. Aptamers’ exquisite binding selectivity for target ligands makes them suitable for a wide variety of applications, including their use as therapeutics[22,25–28], biosensors and detectors [29,30], medical diagnostics[22], protective groups in the synthesis of natural products[31], probes in understanding the molecular basis of disease[32], and excellent recognition domains in naturally occurring riboswitches[33]. Due to their heterogeneous structures when unbound, aptamers also have complex structural landscapes in the absence of ligand, and conformational transitions throughout their landscapes are fundamental for aptamer function. For instance, it has been demonstrated experimentally that the well-characterized theophylline-binding aptamer[34] forms an unstable binding site in the absence of theophylline, and that only ~30–60% of the equilibrium population has a structure conducive to ligand binding[4]. Prior to ligand binding a conformational change is required, in which the unstructured free RNA converts into a binding-competent species. This observation indicates that the theophylline binding mechanism is characterized at least in part by a conformational selection process.

There are more than one hundred published NMR and crystallographic structures of aptamers bound to their target molecules. This wealth of structural data has allowed the ligand-bound structures of aptamers to be well characterized. Unfortunately, as a result of their conformational heterogeneity in the absence of ligand, it is difficult to determine at high resolution the secondary and tertiary structures of unbound aptamers, as well as other types of RNA, using experimental techniques. Structural knowledge of these unbound RNAs generally has to be inferred from indirect experimental methods, such as kinetic assays, or from methods like RNA chemical footprinting[35,36], which yield only ensemble averages for molecules with multiple states. Thus, there is only limited knowledge of aptamer structures in the unbound state. However, characterizing the conformational ensemble of unbound aptamers at atomic resolution would allow us to better understand features of their structural landscapes that are currently difficult to probe, for example, the number and relative populations of equilibrium conformational states, secondary and tertiary structures that characterize these states, and the free energy barriers that separate the states. A better understanding of these features would help to further elucidate the structural and dynamical basis of aptamer function, including the structural roles played by divalent ions and ligand binding mechanisms. Ligand binding mechanisms to aptamers, in particular, remain relatively poorly understood yet are critical for full exploitation of aptamers as therapeutics[37] and in other applications.

In order to characterize at atomic resolution the conformational landscape of an unbound model RNA aptamer, we applied 21 μsec of molecular dynamics (MD) simulations and Markov state modeling to the 33-nucleotide theophylline-binding aptamer in the absence of theophylline. This well-characterized aptamer binds to theophylline with a measured *K*_d_ of ~0.3 μM while discriminating ~10,000-fold against the structurally similar compound caffeine[34]. We report essential features of the modeled aptamer landscape, namely the secondary and tertiary structures and equilibrium populations of kinetically connected metastable conformational states, along with the timescales and free energy barriers of conformational transitions between these metastable states. Moreover, we present the complete binding pathway of theophylline, including the order and nature of conformational transitions in the binding site that lead from the binding-incompetent free RNA to the fully bound RNA observed in the aptamer’s solution NMR structure[38]. Together, our findings suggest that the theophylline binding mechanism involves conformational selection before ligand binding followed by additional conformational rearrangement of the binding pocket after ligand binding. This observation of a subsequent induced-fit step bolsters earlier conjecture that the slow association rate constant of theophylline may be explained by conformational rearrangement in the binding site after initial ligand association[4]. Finally, we show that the presence of Mg^2+^ ions at 10 mM concentration shifts the population of unbound RNA from binding-incompetent to binding-competent states over twofold relative to when Mg^2+^ is absent. Combined with the results of our simulations of theophylline unbinding, this finding supports a prior hypothesis[39] that the Mg^2+^-induced population shift, rather than any intrinsic interactions between Mg^2+^ and theophylline, accounts for altered theophylline binding kinetics in the presence of Mg^2+^. The methods we use to characterize the unbound state of this model RNA can be applied to other RNA molecules of interest.

## Computational methods

### General molecular dynamics simulation parameters

All MD simulations in this paper were performed using the Gromacs 4.6 software[40,41] in conjunction with the AMBER99SB force field[42,43], applying 2 fs time steps, particle mesh Ewald periodic boundary conditions[44], and 1.0 nm non-bonded cutoffs. Bonds were constrained using the LINCS algorithm[45]. Temperature coupling was carried out using the velocity-rescaling thermostat of Bussi *et al*[46]. Simulations in the NPT ensemble used the Parrinello-Rahman pressure coupling scheme[47,48] with a barostat relaxation time of 2.0 ps at a pressure of 1 atm.

#### Replica-exchange molecular dynamics to generate distinct starting RNA conformations

Since RNA molecules have rugged free energy landscapes[49–51], launching multiple MD simulations of an RNA molecule from a single starting conformation is likely to lead to poor sampling of conformational space. Therefore, prior to conducting production MD simulations of the unbound theophylline aptamer, we attempted to generate a diverse set of starting conformations of the RNA located at different points on the molecule’s energy landscape. This sampling was accomplished by using replica-exchange molecular dynamics (REMD)[52] simulations of the unbound aptamer over a wide range of temperatures, allowing barriers in the free energy landscape to be surmounted more effectively. The temperatures for REMD were distributed exponentially such that the expected acceptance probability was 20%, as calculated using an online temperature generator for REMD simulations (http://folding.bmc.uu.se/remd/)[53]. The server yielded 32 replica temperatures over a range of 295–406 K, corresponding to an average temperature spacing of ~3.5 K between replicas.

The starting structure of the unbound aptamer was taken as the first conformer of the solution NMR structure of the aptamer bound to theophylline (PDB entry 1EHT)[38]. After the bound theophylline molecule was removed manually, the resulting ligand-free RNA structure was minimized in vacuo by 100 steps of steepest-descent minimization. The minimized structure was solvated in a dodecahedral box containing 9117 TIP3P water molecules[54] such that all RNA atoms were at least 1.1 nm from the box edge. Thirty-two Na^+^ ions were added to the system to neutralize the overall system charge. A 10 mM Mg^2+^ ion concentration was generated by adding two Mg^2+^ ions and four Cl^−^counterions. The whole system was then minimized for 1000 steps of steepest-descent minimization. Solvent molecules and ions were gradually heated from 200 K to 298 K in the NVT ensemble over the course of 1 ns, while position restraints of 1000 kJ mol^–1^ nm^–2^ were applied to all RNA atoms. The solvent was then equilibrated in the NPT ensemble at 298 K for 1 ns; the same position restraints on all RNA atoms were maintained. Finally, 32 replicas of the solvent-equilibrated system were gradually heated or cooled from 298 K to each of the 32 REMD temperatures (ranging from 295–410 K) in the NPT ensemble over 3 ns without applying any position restraints, followed by an additional 2 ns of unrestrained simulation at the final temperatures. After the replicas were heated to their final temperatures, a 50-ns production REMD simulation was performed in parallel for each replica, with replica exchange being attempted every 500 steps (1 ps). Structures were saved for analysis every 1 ps. We note that we did not attempt to reach converged sampling results via REMD, which would presumably require much longer REMD simulations. Rather, our aim was simply to generate a wider range of starting conformations for our single-temperature production simulations than would be possible when starting from a single experimental structure.

To extract a set of dissimilar conformations for the RNA replica at 298 K, the replica’s 50-ns REMD production trajectory was clustered into thirty clusters based on the root-mean-square deviation (RMSD) of the RNA main chain non-hydrogen atoms relative to those of the starting NMR structure. Clustering was performed using the *k*-means algorithm in the R statistical package (http://www.R-project.org/). Structures from the time points corresponding to the thirty cluster centroids were extracted from the trajectory and used as starting conformations for the subsequent set of production simulations.

### Production simulations of unbound aptamer

We performed 23 successive batches of production MD simulations of the unbound RNA aptamer in the NPT ensemble at 298 K. Each batch consisted of 30 parallel 30-ns simulations, yielding 900 ns per batch and ~21 μs of total production simulation time. RNA conformations from all MD trajectories were saved for analysis every 100 ps.

To begin the first batch of simulations, the thirty RNA conformations from the REMD trajectory at 298 K corresponding to the cluster centroids described previously were used as starting conformations. Initial atomic velocities for each simulation were randomly assigned from a Maxwell distribution at 298 K.

Starting conformations for each subsequent batch of MD simulations were selected using the fluctuation amplification of specific traits (FAST) goal-oriented sampling method recently introduced by Zimmerman and Bowman[55]. The FAST method enhances sampling of conformational space by initiating successive batches of simulations such that the starting points for each batch are chosen from the set of all previously sampled conformations based on a reward function. This reward function calculates the relative probability that simulations initiated from different conformations will discover new conformations that minimize or maximize a user-defined molecular property of interest, for example, RMSD relative to a reference structure, molecular surface area, free energy, etc., provided that the property follows a gradient.

In the present work, the RMSD of all RNA non-hydrogen atoms relative to the starting bound-state NMR structure was chosen as the molecular property *f* whose value we sought to maximize. By maximizing this RMSD, we hope to efficiently explore conformations that are dissimilar to the experimental bound-state structure. Starting conformations for each batch of simulations (other than the first batch) were chosen using the following algorithm[55]:

(i) Cluster all MD simulation conformations sampled so far into 10 discrete states and select the cluster centroids as representative state structures. Ten was chosen as the number of clusters in order to yield an average of three simulations started from each representative structure in the upcoming batch of 30 simulations (step iii).
(ii) For each of the 10 representative state structures, calculate a reward function
  $$r_{f}(i)=\bar{f}(i)+a\bar{y}(i)$$ (1)
  where *i* is the state, $\bar{f}(i)$ is a directed component favoring states that optimize the structural property *f* of interest (in this case the non-hydrogen-atom RMSD relative to the original NMR structure), $\bar{y}(i)$ is an undirected component that favors states that have been poorly sampled so far compared to other states, and *a* is a parameter that dictates the relative importance of the directed and undirected terms. For a property that one wishes to maximize, as we wish to maximize RMSD, the value of $\bar{f}(i)$ is given as
  $$\bar{f}(i)=\frac{f(i)–f_{\text{min}}}{f_{\text{max}}–f_{\text{min}}}$$ (2)
  where *f(i)* is the value of *f* for state *i*, and *f*_max_ and *f*_min_ are the maximum and minimum values of the property of interest, respectively, over all previously sampled conformations. The value of $\bar{y}(i)$, which is applied to favor poorly sampled states, is computed as
  $$\bar{y}(i)=\frac{C_{\text{max}}–C_{i}}{C_{\text{max}}–C_{\text{min}}}$$ (3)
  where *C*_i_ is the number of observations of state *i*, and *C*_max_ and *C*_min_ are the maximum and minimum number of observations of any state, respectively. A value of *a* = 1 was chosen in order to give equal weighting to the directed and undirected terms. Previous work has shown that *a* values ranging from 0.5 to 1.5 often give similar results[55].
(iii) Start a new batch of 30 simulations, using as starting conformations the cluster representatives from the ten clusters in step i, such that the number of simulations launched from each state is proportional to the state’s reward function. Initial atomic velocities were randomly assigned from a Maxwell distribution at a temperature of 298 K for each simulation.
(iv) Repeat steps i-iii until a desired cumulative sampling time has been reached. We performed batches until >20 μs of total simulation time had been achieved, requiring 23 batches.

### Markov state modeling of unbound aptamer simulation data

Markov state models (MSM) are increasingly used to analyze large volumes of MD simulation data to extract long-timescale kinetic information from many short MD simulation trajectories[56–61]. We used the MSMBuilder2 software package[60] to construct an MSM based on ~210,000 RNA conformations taken from the ~21 μs of simulation data. These conformations were clustered into 5000 microstates using the hybrid k-centers/k-medoids clustering method[60] based on the RMSD of all non-hydrogen atoms of the eight nucleotides whose bases are located within 0.8 nm of the bound theophylline in the NMR structure (residues 6, 7, 8, 22, 23, 24, 26, and 28). These residues constitute the theophylline binding site. The number of transitions between microstates at an interval of a certain lag time is counted, and the count matrix is then symmetrized and normalized to obtain the transition probability matrix (**T**). The Markov time, the time scale at which the model is Markovian, is revealed by examining the implied time scales at different lag times. At a given lag time *t*, the implied time scale can be calculated as

$$k(t)=\frac{–t}{\text{ln}[m(t)]}$$ (4)

where *k(t)* is the implied time scale and *m(t)* is an eigenvalue of the transition matrix **T**(*t*). If the model is Markovian at lag time *t*, the implied time scales should remain constant when using longer lag times. The minimum lag time yielding a Markovian model was determined as 20 ns, and this lag time was used to build the microstate MSM.

To make interpretation of the microstate model easier, we coarse-grained the 5000-microstate MSM into a macrostate model containing a small number of macrostates. In order to determine a suitable number of macrostates, we used the Bayesian agglomerative clustering engine (BACE)[62]. The calculated Bayes factor as a function of the number of macrostates was examined, and a number of eight macrostates was the smallest number that immediately preceded a large increase in the Bayes factor. Accordingly, we coarse-grained the microstate model into eight macrostates using BACE. The population of each macrostate was calculated as the sum of the equilibrium populations of microstates belonging to the macrostate. The mean first passage time (MFPT) between each pair of states in the coarse-grained model was calculated using the *CalculateMFPTs* tool implemented in MSMBuilder2.

We assessed whether the macrostate and microstate MSMs for the unbound RNA meet the Markov assumption by testing whether they satisfy the Chapman-Kolmogorov equation. Specifically, we checked whether the approximation

$${\left[T(t)\right]}^{k}\approx T(kt)$$ (5)

holds within the limits of statistical uncertainty. **T**(*t*) is the transition matrix estimated from the MD trajectory data at lag time *t* (the MSM), and **T**(*kt*) is the transition matrix estimated from the same trajectory data at longer lag times *kt*. To perform this check, we applied the method described by Prinz *et al*. [63], according to which one compares the probability *p*MSM(*A*, *A*; *kt)* of being in a given set of states *A* at times *kt* as predicted by the MSM and the corresponding probability *p*MD(*A*, *A*; *kt*) computed directly from the MD trajectory data. For each individual state in the macrostate MSM and for a small sample of individual states in the microstate MSM, a separate Chapman-Kolmogorov test was performed. In each test, only the one given macrostate or microstate being evaluated constituted set *A*. The probabilities *p*MD(*A*, *A*; *kt)* and *p*MSM(*A*, *A*; *kt)* were computed using Eqs 62–63 and Eq 64, respectively, of ref. [63]. Uncertainties in *p*MD(*A*, *A*; *kt)* were estimated using Eq 65 of ref. [63]. Plots of *p*MD(*A*, *A*; *kt)* and *p*MSM(*A*, *A*; *kt)* were generated, and an assessment was made of the extent to which values of *p*MSM(*A*, *A*; *kt)* fall within the range of uncertainty of the corresponding values of *p*MD(*A*, *A*; *kt)*.

To estimate uncertainties in calculated MFPTs, 80% confidence intervals of all MFPTs were computed by transition matrix sampling. Specifically, a Metropolis Monte Carlo simulation with one million steps was applied. At each step, the values in the transition matrix were modified using the nonreversible element shift method described by Noé[64]. After every 1000 steps the MFPT vector was recalculated from the most recently modified transition matrix by solving the matrix equation [65]

$$\left(\begin{array}{cccc} p_{11}\text{–}1 & p_{12} & \overset{}{\underset{↽}{⇀}} & p_{1K}\\ p_{21} & p_{22}\text{–}1 & \overset{}{\underset{↽}{⇀}} & p_{2K}\\ & \ddots & & \\ p_{(K\text{–}1)1} & \overset{}{\underset{↽}{⇀}} & p_{\left(K\text{–}1)(K\text{–}1\right)}\text{–}1 & p_{(K\text{–}1)K}\\ 0 & \overset{}{\underset{↽}{⇀}} & 0 & 1 \end{array}\right)\left(\begin{array}{c} x_{1}\\ x_{2}\\ \vdots \\ x_{K\text{–}1}\\ x_{K} \end{array}\right)=\left(\begin{array}{c} \text{–}t\\ \text{–}t\\ \vdots \\ \text{–}t\\ 0 \end{array}\right)$$ (6)

where the matrix on the left side of Eq (6) is referred to as **A**, with rows **a**_i_, the vector on the left side is the set of MFPTs from state *i* to the final state *K*, *t* is the lag time, and the final line is the boundary condition that the MFPT from the final state to the final state must be zero. From the 1000 sets of recalculated MFPT vectors, 80% confidence intervals were computed for each MFPT.

Uncertainties in macrostate populations were likewise estimated by calculating 80% confidence intervals using the same Metropolis Monte Carlo procedure in conjunction with the nonreversible shift element method applied to the transition matrix. A new set of populations was calculated after every 1000 steps by solving for the normalized first left-eigenvector of the most recently modified transition matrix. From the set of recalculated populations 80% confidence intervals were computed.

### Estimating theophylline binding affinities to RNA conformers in each MSM macrostate

In an effort to determine which MSM macrostates are competent for ligand binding, we predicted average theophylline binding affinities to the RNA conformations in each of the eight macrostates in the coarse-grained MSM using molecular docking. The *SaveStructures* tool in MSMBuilder2 was used to save 500 randomly selected conformations from each of the eight macrostates. AutoDock Vina[66] was then used to dock theophylline in the binding site region in all of the 4000 selected RNA conformations. Atomic charges for theophylline and the RNA were generated automatically using the *prepare_ligand4*.*py* and *prepare_receptor4*.*py* tools, respectively, contained in the AutoDock Tools package[67] with their default parameters. The docking region was defined as a cube with sides of length 1.0 nm centered at the center of mass of the bases of RNA residues 6, 7, 8, 22, 23, 24, 26, and 28. All RNA binding site residues were held rigid during docking, while full flexibility was allowed for the theophylline ligand. For each receptor-ligand complex the most negative binding score was tabulated for analysis. For the purpose of comparison, theophylline was likewise docked in its binding site for the first conformer of the NMR structure.

### Simulating experimental structure of RNA-theophylline complex

The theophylline-bound state of the RNA aptamer was modeled to compare the dynamics of the bound state with those of the unbound state. The first conformer of the NMR structure of the aptamer bound to theophylline was used as the starting structure. Force field parameters and a topology file for theophylline were prepared by ACPYPE[68]. Charges were calculated using the ACPYPE default semi-empirical quantum chemistry program[69]. The RNA-theophylline complex structure was solvated in a dodecahedral box containing 8352 TIP3P water molecules in the presence of 10 mM Mg^2+^ and equilibrated using the same protocol as for the simulations of unbound RNA. Following equilibration, 80 separate 10-ns production MD simulations were performed at 298 K, using different initial atomic velocities for each simulation. Structures were saved for analysis every 100 ps.

### Mapping theophylline binding pathway

Mapping the complete theophylline binding pathway to the unbound RNA aptamer can provide insight into the theophylline binding mechanism. We performed this mapping by running multiple sets of MD simulations of theophylline at discrete stages of its binding to the aptamer. The centroid RNA conformation of the most highly populated binding-competent macrostate from the coarse-grained MSM described previously was selected as a representative unbound RNA structure.

#### Mapping diffusion of theophylline into binding site and initial complex formation

The diffusion of theophylline into its RNA binding site and subsequent formation of the initial RNA–theophylline complex were modeled by 16 sets of MD simulations starting from different points along theophylline’s approach pathway and entry pathway into the binding site. The simulation sets had starting conformations with varying distances between theophylline and its binding site. To generate these initial conformations with varying distances, we first performed a single 20-ns MD simulation of theophylline unbinding from this representative RNA structure at 298 K. Force field parameters for theophylline were the same as those used for simulating the RNA–theophylline complex. The starting structure for the unbinding simulation was the bound complex between theophylline and the representative RNA structure as generated by AutoDock Vina. This structure was solvated in the presence of 10 mM Mg^2+^ and equilibrated using the same protocol as for the simulations of unbound RNA. Theophylline unbinding was forced to occur on a short time scale by applying well-tempered metadynamics[70,71] as implemented with the PLUMED 1.3 plugin[72]. Well-tempered metadynamics generates on the fly a history-dependent biasing potential as a function of a set of user-defined collective variables (CV), discouraging the system from becoming trapped in low-energy basins of phase space and allowing long-timescale events, such as ligand unbinding, to be observed within normal simulation timescales. In the present work, we selected as a single CV the distance between the center of mass of theophylline and the center of mass of the binding site residues’ bases. This CV was biased using a starting Gaussian height of 2.0 kJ/mol, Gaussian widths of *σ* = 0.01 nm, a bias factor of 10, and a Gaussian deposition rate of 500 time steps (1 ps). Theophylline completely dissociated from the RNA during the simulation, entering the bulk solvent and ending at a distance > 3.0 nm from its binding site. Sixteen snapshots of the unbinding simulation were taken at approximately equally spaced values of the CV ranging from 0.4 nm to 3.0 nm, providing a continuous set of conformations linking the bound state to the fully unbound state of theophylline.

The 16 snapshots taken from the theophylline unbinding simulation served as starting conformations for 16 sets of unbiased MD simulations of the RNA-theophylline system performed at 298 K. Each simulation set consisted of ten separate 20-ns simulations that began from the same starting structure but had different randomly assigned atomic velocities. Structures were saved for analysis every 20 ps. The average simulation box volume for all RNA-theophylline system simulations is 155 nm^3^, yielding an average theophylline concentration of 10 mM.

An MSM was constructed to analyze the 160,000 conformations taken from the aggregate 3.2 μs of simulation time for the RNA-theophylline system. The conformations were clustered into 1000 microstates using the hybrid k-centers/k-medoids clustering method, applying as a clustering metric vectors containing intermolecular distances between five pairs of atoms located in theophylline and in the RNA binding site (theophylline atom N7—C22 atom N3; theophylline atom N9—U24 atom N3; theophylline atom O6—C22 atom N4; theophylline atom N9—U24 atom O4; theophylline atom O2—C8 atom O4’). Plotting of implied time scales showed Markovian behavior beginning at a lag time of 2 ns, and this lag time was thus selected as the lag time for model building. The 1000-microstate model was subsequently coarse-grained into a MSM with 10 macrostates using BACE. The flux and MFPT between macrostates were calculated using the *FindPaths* and *CalculateMFPTs* tools, respectively, of MSMBuilder2.

#### Mapping conformational rearrangement/induced fit process following initial theophylline binding

In the initial complex formed between the RNA and theophylline, the binding site is in a non-ideal conformation that differs greatly from that of the full-affinity complex seen in the NMR structure. As a result, further structural changes in the binding site must take place. To model these changes, we employed 10 rounds of FAST, with each round consisting of 30 individual 20-ns MD simulations, providing a total of 6 μs of simulation data. The RMSD between the non-hydrogen atoms of the binding site residues relative to those of the first conformer of the NMR structure was used as the structural property to minimize. The discrete conformations and numbers of each conformation to use at the beginning of each FAST round were determined using the protocol outlined earlier.

Following the MD simulations, microstate and macrostate MSMs of the trajectories were generated. Conformations were clustered into 5000 microstates by the hybrid k-centers/k-medoids method using the same RMSD metric as that used for the FAST protocol. A lag time of 5 ns allowed Markovian behavior and was thus used for building the microstate MSM. To determine a suitable number of macrostates into which the microstate MSM could be coarse-grained, a plot of the BACE-generated Bayes factor as a function of number of macrostates was examined. A quantity of six macrostates immediately preceded a large increase in the Bayes factor, and hence the microstate MSM was coarse-grained into a MSM containing six macrostates using BACE. A lag time of 5 ns likewise allowed Markovian behavior for the macrostate MSM and was used as the lag time for model construction.

### Modeling theophylline-bound and unbound RNA aptamer in the absence of Mg^2+^

The theophylline-bound and the unbound RNA aptamer were simulated in the absence of Mg^2+^ in an effort to assess at atomic resolution the effects of Mg^2+^ on RNA dynamics and the basis for the influence of Mg^2+^ on theophylline binding kinetics. The structure of the theophylline-bound aptamer in the absence of Mg^2+^ was prepared from the initial NMR structure and equilibrated using the same procedure as for the theophylline-bound aptamer in the presence of Mg^2+^ but without adding Mg^2+^. After equilibration 80 separate MD simulations of length 10 ns were performed at 298 K, applying different initial atomic velocities for each simulation. MD structures were saved for analysis every 100 ps.

The structure of the unbound aptamer in the absence of Mg^2+^ was prepared starting from the NMR structure after deleting the theophylline. Equilibration was conducted following the same procedure as for the unbound aptamer in the presence of Mg^2+^, with the exception of adding Mg^2+^ to the system. REMD and structural clustering of the replica at 298 K were subsequently performed identically to those described previously. Thirty cluster centroids were used as initial conformations for a batch of 30 MD simulations of length 30 ns at 298 K. Five additional batches of 30 simulations each were subsequently carried out in succession, where starting conformations for each batch were determined using FAST. The RMSD of non-hydrogen atom positions relative to those in the starting structure was used as the metric to maximize. A total of 5.4 μs of simulation time was obtained. MD structures were saved for analysis every 100 ps. The conformational space of the unbound aptamer in the absence of Mg^2+^ was modeled by a MSM. Conformations were first clustered into 5000 microstates based on the RMSD of non-hydrogen atoms in residues 6, 7, 8, 22, 23, 24, 26, and 28 using the hybrid k-centers/k-medoids clustering method, and a MSM was then constructed employing a lag time of 20 ns. The 5000-microstates model was subsequently coarse-grained by BACE into a MSM containing 6 macrostates.

### Predicting RNA–theophylline complex lifetimes

We estimated the kinetics of theophylline unbinding from its RNA binding site in the presence and absence of Mg^2+^ by predicting the lifetime of the RNA–theophylline complex, which is the inverse of the dissociation rate *k*_off_ of theophylline. Starting from the equilibrated NMR structure of the RNA-theophylline complex in the presence of 10 mM Mg^2+^, a set of 40 independent well-tempered metadynamics simulations using PLUMED1.3 was applied to accelerate theophylline unbinding at 298 K. The distance between the center of mass of theophylline and the center of mass of the binding site residues’ bases was chosen as the first CV. The solvent coordination number of theophylline was chosen as the second CV, using as parameters *n* = 6, *m* = 12, *r*_0_ = 0.05 nm, and *d*_0_ = 0.25 nm. The first CV alone was biased using a starting Gaussian height of 2.5 kJ/mol, Gaussian widths of *σ* = 0.05 nm, a bias factor of 10, and a Gaussian deposition rate of 2500 time steps (5 ps). Simulations were stopped once theophylline was unbound and fully solvated, as determined by the theophylline solvent coordination number (second CV) reaching a stable plateau. The unbiased rate of theophylline unbinding was obtained from the biased rate using the method recently introduced by Tiwary and co-workers[73]. Briefly, the acceleration provided by metadynamics for a process such as ligand unbinding can be calculated from the bias deposited throughout the simulation. The acceleration factor *a* is determined by a running average accumulated throughout the course of the metadynamics simulation and is given by

$$a=\langle e^{V(s,t)/k_{B}T}\rangle$$ (7)

where *s* is the biased CV and *V(s*,*t)* is the bias experienced at time *t*[74–76]. The latter is obtained from the COLVAR file produced by PLUMED during the metadynamics simulation. To obtain the unbiased time for theophylline unbinding, the acceleration factor *a*, as calculated at the time when complete unbinding has occurred, is multiplied by the observed simulation time required for this complete unbinding. This process was repeated for all 40 independent metadynamics simulations. The distribution of unbiased unbinding times was analyzed by applying the method described in ref. [77]. This analysis yields an estimate of the characteristic time required for theophylline unbinding, which is equivalent to the RNA–theophylline complex lifetime. The standard error of the computed lifetimes was estimated using the bootstrapping method[78].

This procedure was repeated for the equilibrated NMR structure of the RNA-theophylline complex in the absence of Mg^2+^ and for a single structure taken from the aggregate simulation data for the unbound RNA in the absence of Mg^2+^. The latter structure was obtained by clustering all the saved conformations from the set of Mg^2+^-free simulations of unbound RNA into a single cluster and selecting the centroid structure, followed by docking of theophylline into the binding site of the centroid structure using AutoDock Vina.

### RNA secondary structure and base stacking calculations

RNA secondary structure and base stacking calculations were performed by the 3DNA-DSSR suite of programs[79] for analysis of nucleic acid structures.

### RNA images

All RNA tertiary structural images were produced using UCSF Chimera[80].

## Results and discussion

In the following sections, we comment on validation of the Markov state model (MSM) for MD simulations of the unbound RNA aptamer. Then we characterize the conformational landscape of the unbound aptamer by analyzing both global structure and local structure involving the theophylline binding site. We similarly characterize the conformational landscape of the bound aptamer and highlight its similarities and differences relative to the unbound-state landscape. Moreover, we map the complete binding pathway of theophylline and discuss its modeled binding mechanism. Finally, we analyze the role that Mg^2+^ plays in stabilizing the aptamer and the structural basis for how Mg^2+^ affects the kinetics of theophylline unbinding.

### MSM validation for simulations of unbound RNA

MSMs are kinetic network models that model conformational dynamics of biomolecules as transitions between metastable states[56–61,81]. A major advantage of applying MSMs in conjunction with MD simulations is that MSMs offer an efficient sampling strategy permitting many short MD trajectories to be used to sample transitions between metastable states, while MSM networks describe long-timescale dynamics and equilibrium properties. The use of MSMs thus often makes it unnecessary to conduct long-timescale simulations, instead allowing brief simulations to be run in parallel. MSM methods have recently proven highly useful for modeling conformational dynamics of biomolecules on long timescales[55,82–85], including protein folding simulations on the millisecond timescale[86]. Consequently, we generated both microstate and macrostate MSMs to probe the conformational landscape of the unbound RNA aptamer at 298 K, including the distinct RNA conformations that characterize metastable states and the kinetics and energetics of transitions between states.

**Fig 1:**
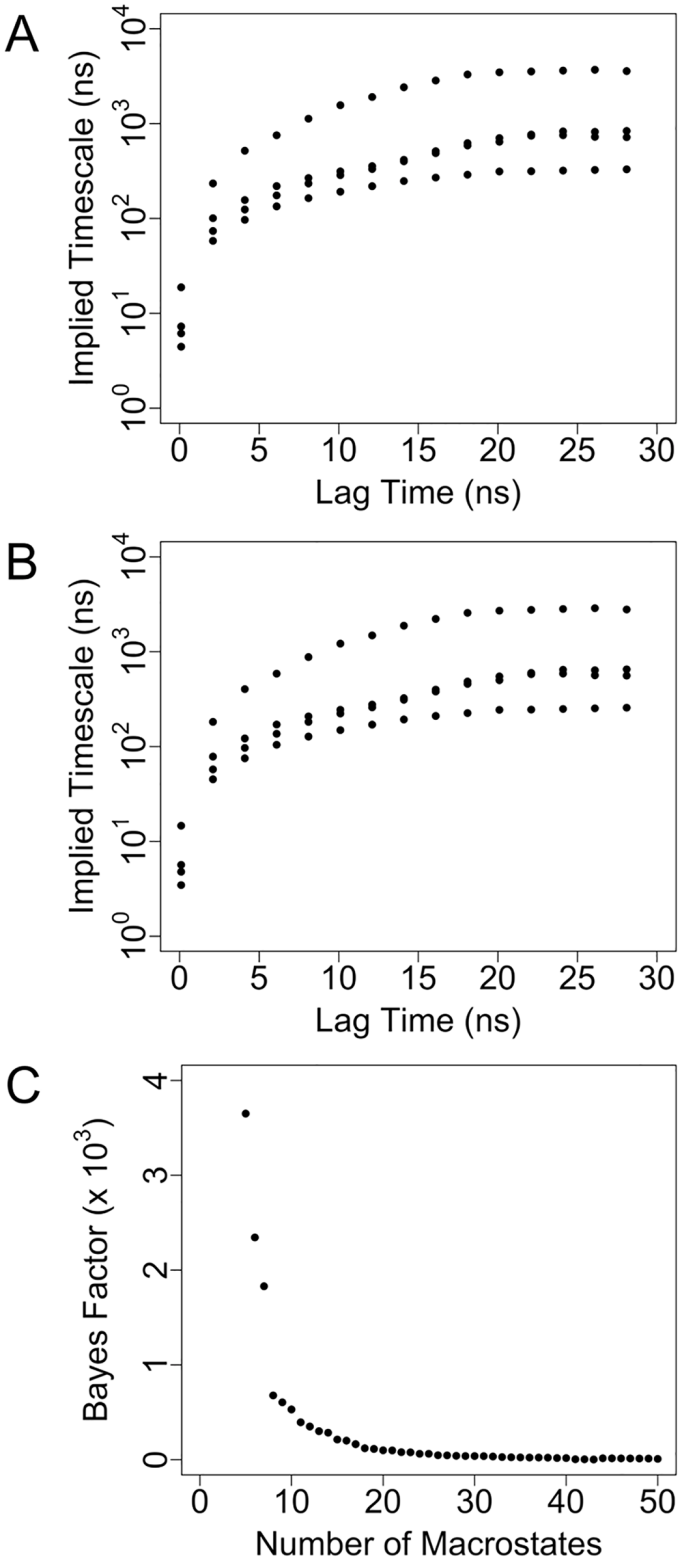
Implied timescales of Markov state models of unbound RNA. (A) Implied timescale plot for the 5000-state microstate MSM. (B) Implied timescale plot for the 8-state macrostate MSM. (C) Bayes factor as a function of number of coarse-grained macrostates. The smallest number of macrostates immediately preceding a large increase in the Bayes factor (8) was selected as the number of macrostates to include in the macrostate MSM.

We have validated that our MSMs for the aggregate ~21 μs of MD simulation data for the unbound RNA are Markovian in nature. Adequate sampling is essential for determining valid kinetic and equilibrium information from molecular simulations. MSMs allow extraction of such information from large numbers of simulations that are individually much shorter than the timescales of the phenomena being monitored, but Markovian behavior of the constructed model must be confirmed. In MSM validation, a requirement for Markovian behavior is that the MSM satisfy the Chapman-Kolmogorov equation and that implied timescales remain constant at different lag times. We validated the latter by observing the implied timescale plot at different lag times. The implied timescales for the 5000-microstate model cease to change after a lag time of ~20 ns (Fig 1A), which was thus used to construct the microstate MSM. In the present study the quantitative properties, including mean first-passage times (MFPTs), are computed from the microstate MSM. We similarly evaluated the Markovian behavior of the coarse-grained 8-macrostate model that was generated by BACE, and the implied timescales likewise remain constant after a lag time of ~20 ns (Fig 1B), which was applied to construct the macrostate MSM. The selected number of eight macrostates was based on the plot of the computed Bayes factor as a function of number of macrostates, which shows a marked increase between eight and seven macrostates (Fig 1C).

Additionally, we evaluated the Markovianity of the macrostate and microstate MSMs of the unbound RNA by applying a more rigorous test of whether the models satisfy the Chapman-Kolmogorov equation. For the macrostate MSM, the probabilities *p*MSM(*A*, *A; kτ*) of being in each macrostate at times *kτ* (ranging from 1 to 3) as predicted by the MSM were compared with the corresponding probabilities *p*MD(*A*, *A; kτ*) as calculated directly from the trajectory data (S1A Fig). The two probabilities agree reasonably well for many values of *kτ* for macrostates 1, 4, 7 and 8, with the values of *p*MSM(*A*, *A; kτ*) occurring within the range of uncertainties in *p*MD(*A*, *A; kτ*) (error bars in S1A Fig), but the probabilities agree less well for the remaining macrostates. For the microstate MSM, due to the low individual populations of most microstates, the uncertainties associated with *p*MD(*A*, *A; kτ*) are generally larger than those observed for the macrostate MSM. For a small, randomly chosen sample of analyzed microstates (1, 100 and 1000), there is agreement within error between *p*MD(*A*, *A; kτ*) and *p*MSM(*A*, *A; kτ*) for a majority of *kτ* values, while for microstate 5000 there is poorer agreement (S1B Fig). It should be noted that, although the maximum *kτ* value of 3 used here is not particularly large for a thorough application of the Chapman-Kolmogorov test, it is the greatest value that can be applied since the original *τ* is 10 ns and the individual simulations are of length 30 ns. Overall, the Chapman-Kolmogorov test results do not indicate strong Markovian behavior. Nonetheless, as is frequently noted by other authors, the invariability of the implied timescales beyond the chosen value of *τ* strongly suggests that the overall behavior of the model is reasonable[87], despite some individual states not being perfectly Markovian.

### Analysis of conformational landscape of unbound RNA

**Fig 2:**
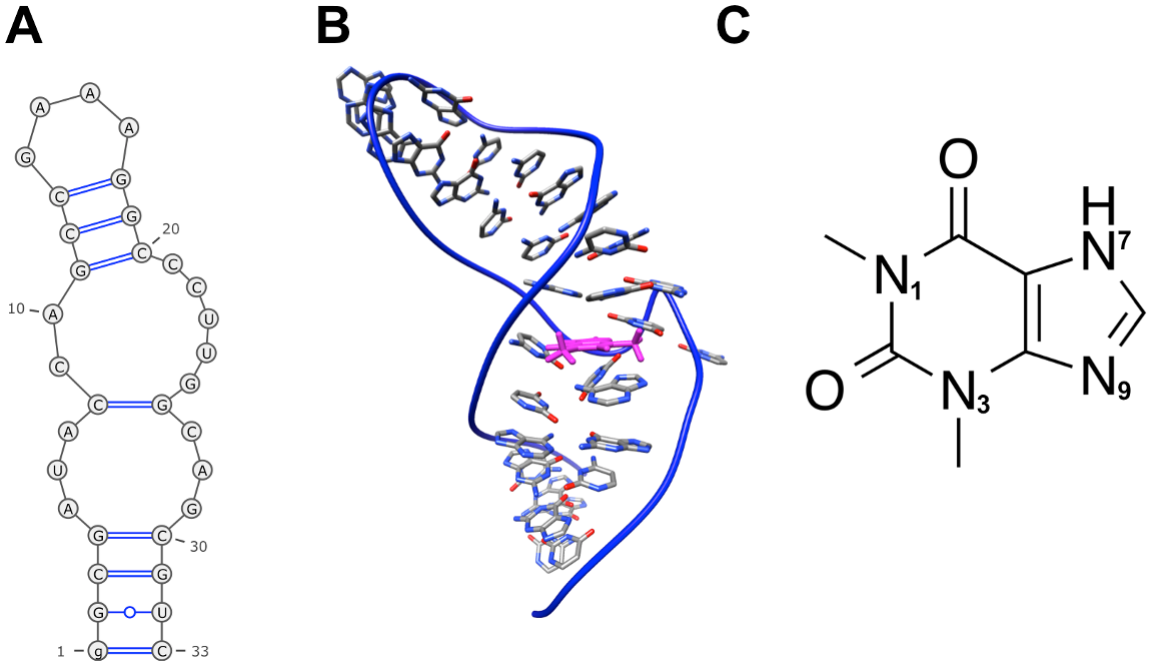
Solution NMR structure of theophylline aptamer in the bound state. (A) Secondary structure. (B) Tertiary structure. The bound theophylline molecule is colored magenta. (C) Theophylline structure. Depicted RNA structures are based on the first conformer of PDB entry 1EHT. Secondary structure diagram was generated by the RNApdbee webserver[88].

**Fig 3:**
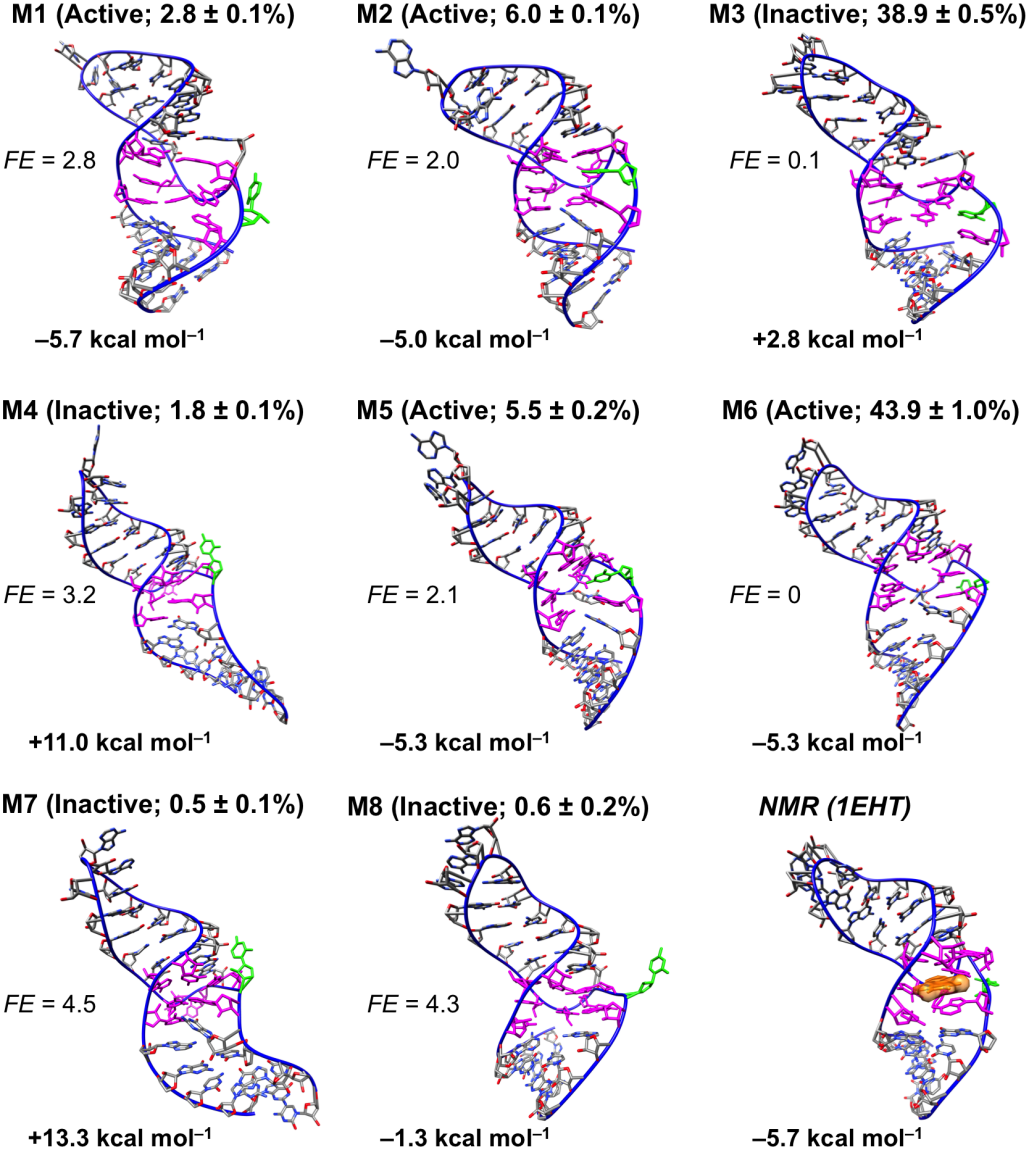
Conformational diversity of unbound RNA in the presence of 10 mM Mg^2+^. Shown are centroid structures of the eight macrostates (M1-M8) taken from the macrostate MSM constructed from 21 μs of MD simulation data. Within parentheses, binding-competent and binding-incompetent macrostates are labeled *active* and *inactive*, respectively, and relative populations of macrostates are indicated. Free energies *FE*_*i*_ (units of *kT*) of macrostates relative to *FE*_*ref*_, the free energy of the most populated macrostate (macrostate 6, labeled M6), are listed. *FE*_*i*_ values are computed as *FE*_*i*_ = –ln(*p*_*i*_/*p*_*ref*_), where *p*_*i*_ and *p*_*ref*_ are respectively the relative populations of the *i*th macrostate and the most populated macrostate. Average AutoDock Vina scores for theophylline docking to 500 randomly sampled conformations from each macrostate are listed below the RNA images. Nucleotides defining the theophylline binding site are colored magenta, and C27 is colored green. To enable comparison with the experimental bound-state structure, the first conformer of the solution NMR structure (PDB entry 1EHT) is also shown; the molecular surface of bound theophylline is colored orange.

In the presence of bound theophylline, the theophylline aptamer forms a stable structure with well conserved secondary and tertiary structure (Fig 2). However, as shown by the modeling data presented here and by prior NMR studies, in the absence of theophylline, the RNA aptamer has a complex conformational landscape, exploring a diverse range of three-dimensional conformations. Our macrostate MSM indicates that two of the kinetically stable macrostates dominate the overall conformational landscape of the unbound aptamer in terms of relative populations (Fig 3). These two most populated macrostates, states 3 and 6, have relative populations of 43.9% and 38.9%, respectively, together accounting for approximately 83% of the totality of microstate populations that were coarse-grained into the macrostate MSM. The remaining six macrostates are much less populated, with relative populations ranging from 0.5% to 6.0%.

**Fig 4:**
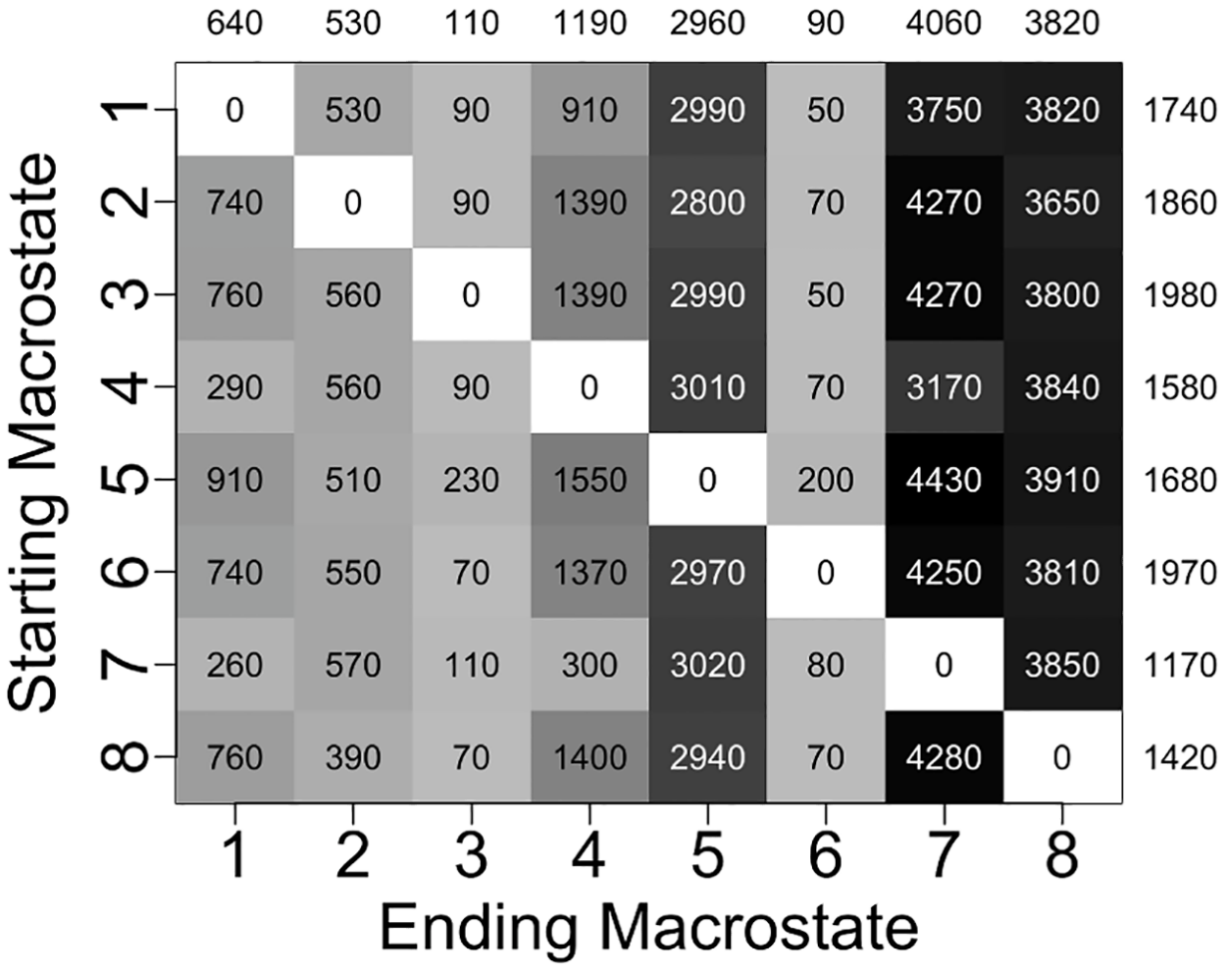
Computed mean first passage times (MFPTs) between different macrostates. Average MFPTs into each ending macrostate are listed above the columns, and average MFPTs out of each starting macrostate are listed to the right of the rows. All times are in terms of nanoseconds.

The mean time required for the RNA to reach a given ending macrostate *j* from a different starting macrostate *i*, referred to as the mean first passage time (MFPT_*ij*_) for that transition, ranges from 50 ns to 4430 ns and has an average value of 1670 ns for all 56 possible transitions (Fig 4). Transitions to the two most populated states (states 6 and 3) occur most quickly, with average MFPTs of 90 ns and 110 ns, respectively, whereas transitions to the two least populated states (states 7 and 8) occur most slowly, with average MFPTs of 4060 ns and 3820 ns, respectively. Similarly, transitions out of the two most populated states take place on the slowest timescale (average MFPTs of 1970 ns and 1980 ns), while transitions out of the two least populated states take place on the fastest timescale (average MFPTs of 1170 and 1420 ns).

**Fig 5:**
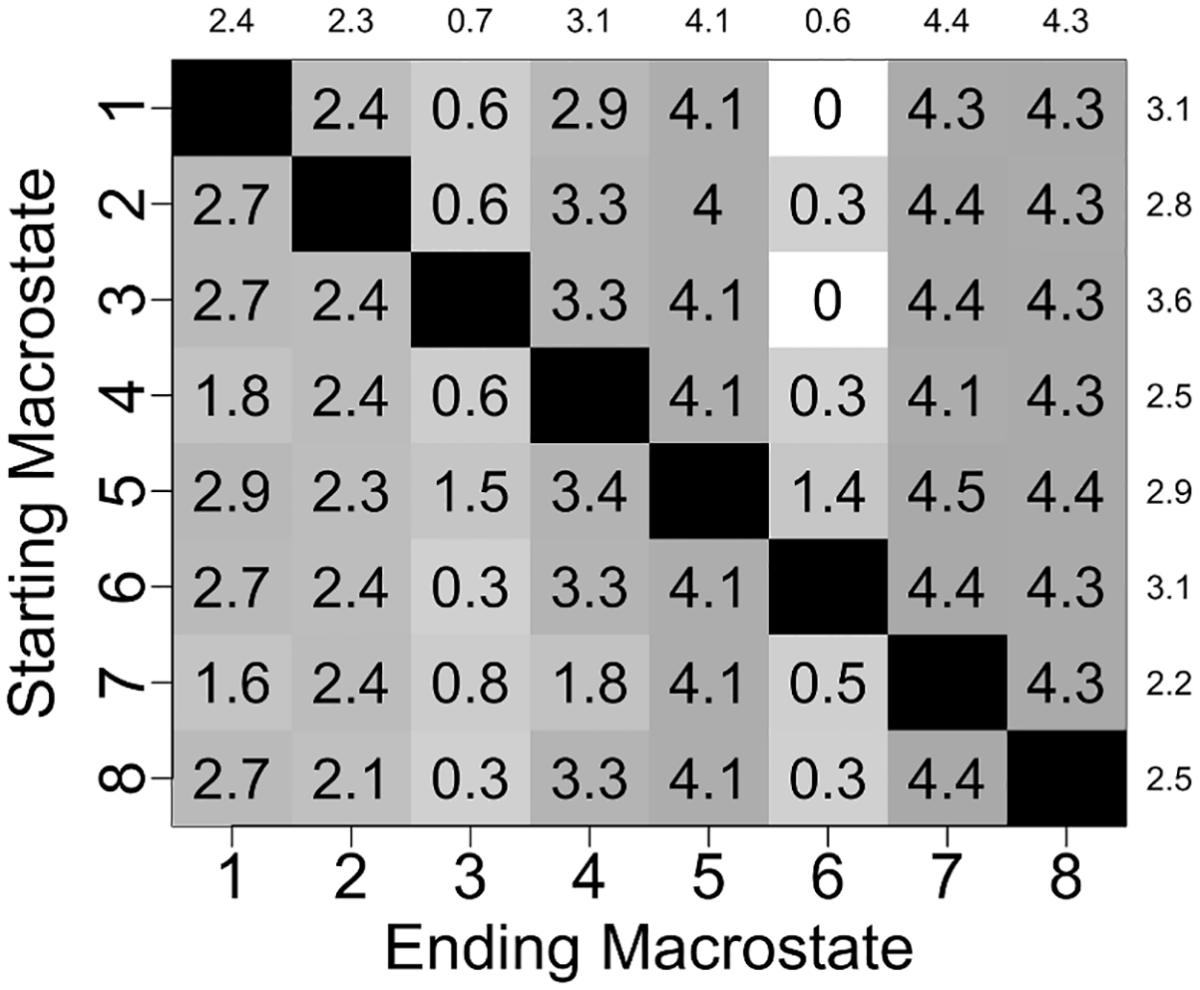
Free energy barriers separating MSM macrostates relative to the two lowest free energy barriers. Average relative free energy barriers for transitions into each ending macrostate are listed above the columns, and average relative free energy barriers for transitions out of each starting macrostate are listed to the right of the rows. All energies are in units of *kT*. (The two lowest free energy barriers correspond to transitions from state 1 to state 6 and from state 3 to state 6.)

The value of MFPT_*ij*_ is inversely proportional to *k*_ij_, where *k*_*ij*_ is the rate constant for the transition between macrostates *i* and *j*. Since rate constants are proportional to the exponential of free energy barriers, assuming Arrhenius kinetics, differences in MFPTs reflect differences in heights of free energy barriers between macrostates. Transitions from state 1 to state 6 and from state 3 to state 6 are the fastest transitions, both having an MFPT of 50 ns, and thus the free energy barriers for these two transitions are expected to be the lowest barriers separating macrostates in the free energy surface of the unbound RNA. Heights of free energy barriers for all other macrostate transitions relative to the lowest barriers can be estimated directly from the set of MFPT values. As shown in Fig 5, the largest free energy barrier is ~4.5 *kT* higher than the lowest barriers. This transition corresponds to the transfer from state 5 to state 7, the least populated state featuring the highest relative free energy.

Four of the eight macrostates are amenable to theophylline binding. To estimate which macrostates are competent for theophylline binding, we attempted to dock theophylline in its binding site in 500 RNA conformations randomly taken from each macrostate. Since theophylline docks in its binding site in the NMR structure with an AutoDock Vina binding score of –5.7 kcal mol^–1^, we selected a binding score of –5.0 kcal mol^–1^ as a cutoff separating binding-competent and binding-incompetent states. A macrostate is designated as binding-competent (active) if its average theophylline docking score is ≤ –5.0 kcal mol^–1^ and is otherwise designated as binding-incompetent (inactive). As shown in Fig 3, only macrostates 1, 2, 5, and 6 are binding-competent, having average docking scores of –5.7 kcal mol^–1^, –5.0 kcal mol^–1^, –5.3 kcal mol^–1^, and –5.3 kcal mol^–1^, respectively. These four macrostates collectively account for 58% of the relative population. This proportion of binding-competent states agrees well with the experimental observation that 33–62% of the unbound aptamer is in a conformation that permits theophylline binding[4].

**Fig 6:**
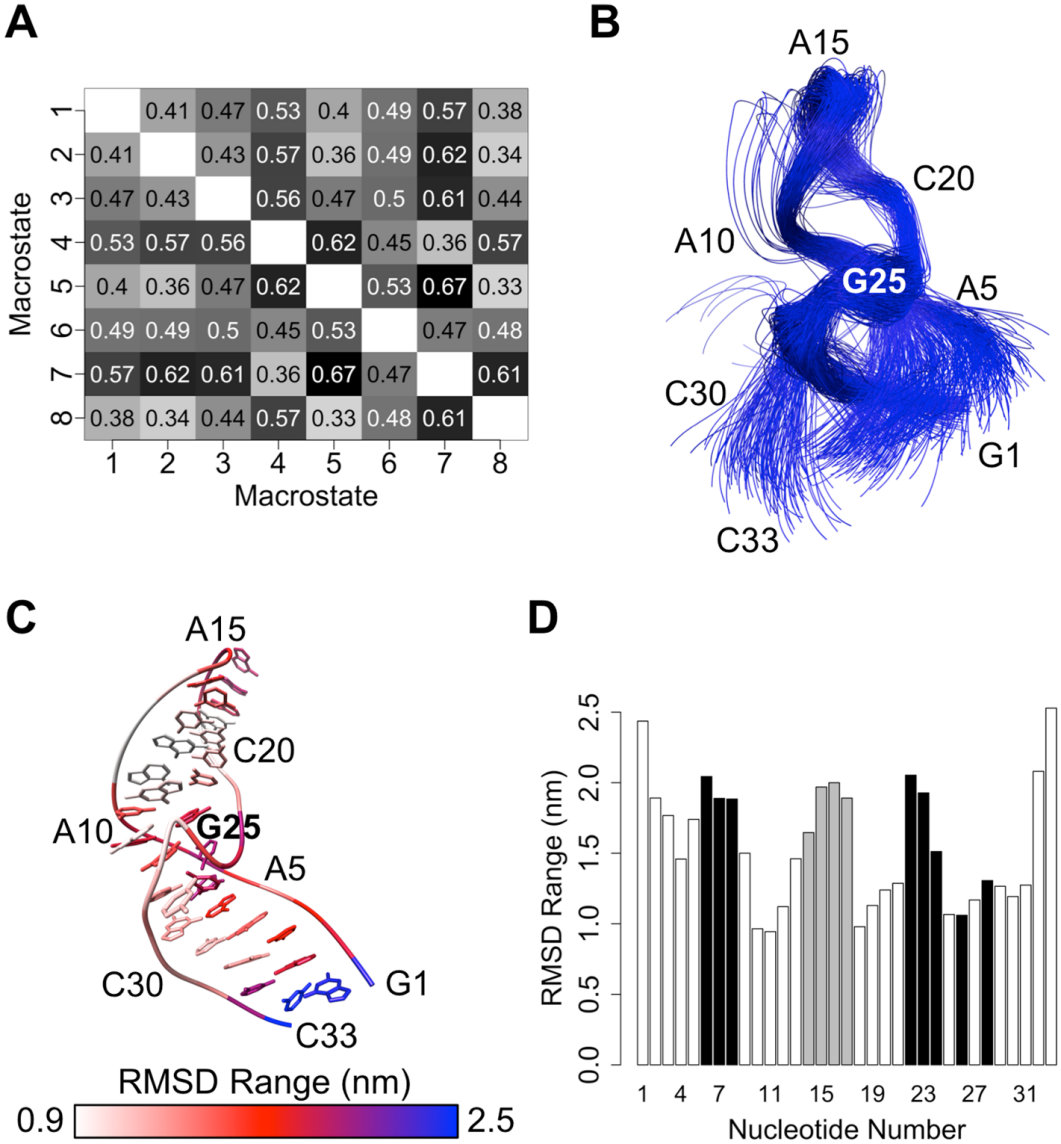
Variability of RNA tertiary structure in the unbound state. (A) All-versus-all root-mean-squared deviation (RMSD) chart for pairs of macrostates. Average main chain RMSD values (in nm) for all conformations within each macrostate pair are listed. (B) Main chain overlay of 50 conformations selected randomly from each macrostate. (C) Main chain RMSD ranges of individual nucleotides relative to the reference NMR structure after main chain fitting. The NMR structure is depicted, and per-nucleotide RMSD ranges are colored by nucleotide. (D) Bar plot of ranges of RMSD values of individual nucleotides relative to the NMR structure after main chain fitting. Bars corresponding to nucleotides in the theophylline binding site and nucleotides of the GAAA tetraloop are colored black and gray, respectively.

The unbound RNA tertiary structure undergoes significant conformational changes between the eight macrostates (Figs 3 and 6). To structurally characterize the major free energy basins in the conformational landscape, we analyzed both the tertiary and secondary structures of conformers within each macrostate. The RNA main chain (atoms P, O5’, C5’, C4’, C3’, and O3’) adopts a wide range of tertiary structures throughout the 21 μs of simulations. These tertiary structures have global main chain RMSD values relative to the NMR structure that range from 0.2 nm to 1.4 nm, with an average value of 0.50 nm. The main chain conformation often varies widely also between macrostates (Fig 6A). For example, the conformers in states 5 and 7 have an average main chain RMSD of 0.67 nm relative to one another, while the conformers in states 4 and 5 have a similar average main chain RMSD of 0.62 nm. In contrast, states 5 and 8 are more similar to one another in terms of main chain conformation, with an average RMSD of only 0.33 nm. As shown in Fig 6B–6D, not only does the global main chain conformation adopt heterogeneous structures, but different regions of the RNA structure also show varying degrees of flexibility and ranges of motion. The G1 and C33 termini undergo the largest range of motions, as their individual main chain RMSD values relative to the NMR structure span a wide interval of ~2.5 nm. Similarly, the GAAA tetraloop (nucleotides 14–17) and five of the nucleotides defining the theophylline binding site (nucleotides 6, 7, 8, 22 and 23) are highly mobile, having per-residue RMSD value ranges of ~2.0 nm.

Similarly to the GAAA tetraloop and a portion of the binding site, C27 is dynamic and explores a wide range of conformations. The C27 base alternates between being buried in the theophylline binding site in macrostates 2, 3 and 5, partially exposed to solvent in macrostates 4 and 7, and completely exposed to solvent in macrostates 1, 6 and 8 (Fig 3). For the unbound-state simulations, the average solvent-accessible surface area (SASA) of the C27 base is 0.65 ± 0.20 nm^2^ (mean ± standard deviation), whereas for the NMR structure of the theophylline-bound state the corresponding SASA is 0.99 nm^2^. Moreover, the C27 base forms base stacking interactions with nucleotides both above and below the plane of its base in 35% of the sampled unbound conformations. These observations are consistent with prior experimental work showing that C27 is predominantly buried and participates in extensive base stacking interactions when the RNA is unbound but shifts outward into solution when theophylline binds[38].

**Fig 7:**
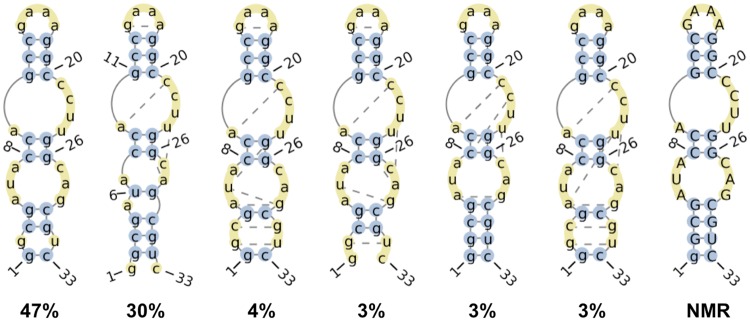
Six most frequently observed RNA secondary structures in the absence of bound theophylline. Numbers denote proportions of all analyzed simulation snapshots in which the RNA adopts the depicted secondary structures. Bases that participate in canonical and non-canonical base pairing interactions are depicted in blue circles and yellow circles connected by dotted lines, respectively. Unpaired bases are depicted in unconnected yellow circles. The secondary structure of the bound-state NMR structure is shown on the far right. Secondary structure diagrams were generated by the RNApdbee webserver[88].

In the absence of bound theophylline, the secondary structure of the RNA also varies significantly. Throughout the 21 μs of simulation time, 39 unique secondary structures are observed (Fig 7). The stem containing the G11-C20, C12-G19, and C13-G18 canonical Watson-Crick (WC) base pairs, the stem containing the C8-G26 and C9-G25 WC base pairs, and the G4-C30 WC base pair remain intact in all observed secondary structures. However, in contrast to the RNA secondary structures for the ten conformers of the bound-state NMR structure, the G1-C33, G2-U32, and C3-G31 WC base pairs are formed only in a portion of the simulated conformations of the unbound RNA. The G2-U32 and C3-G31 base pairs frequently rearrange such that they adopt a non-WC conformation. Of the 46 hydrogen bonds occurring between bases in the NMR structure, an average of only 30 ± 2 hydrogen bonds are formed in the aggregate simulation time, further indicating that removal of theophylline perturbs the native (bound-state) secondary structure. Moreover, when the RNA is not bound to theophylline, a non-native base pair forms in the theophylline binding site between the bases of U6 and G29 in ~40% of conformations. This base pairing interaction helps to close the binding site to make the RNA incompatible with ligand binding (see below).

**Fig 8:**
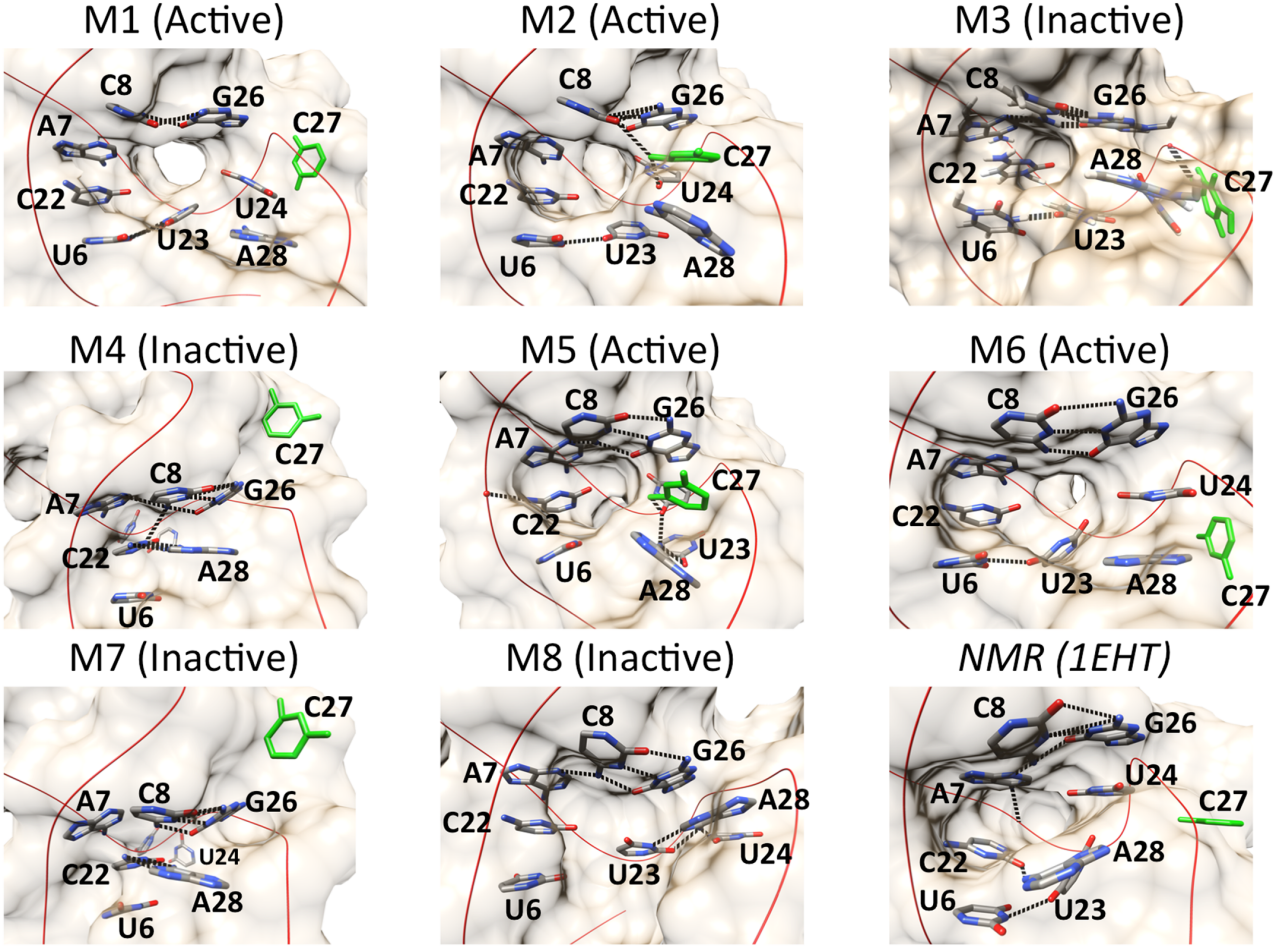
Spatial arrangements of the bases that form the theophylline binding site in macrostates 1–8. The base of nucleotide C27 is colored green. Hydrogen bonds are depicted as dotted lines.

The theophylline binding site is destabilized and takes on multiple heterogeneous conformations among the eight macrostates that the unbound RNA explores. Several of the residues that form the binding site adopt significantly different spatial arrangements relative to one another in each macrostate (Fig 8). Although C8 and G26 consistently form a native WC base pair and U6, C22, and A7 consistently form base stacking interactions, the bases of U23, U24, and A28 are inconsistent in their relative positions. In macrostates 1 and 6, for instance, U24 and A28 form base stacking interactions with each other, whereas in macrostates 2 and 7 they are separated by 0.9 nm and 1.7 nm, respectively. Similarly, the base of U23 pairs with the base of U6 in macrostates 1, 2, 3, and 6, as well as in the NMR structure, but in macrostates 4 and 7, it swings outward into solution to a position ~1.5 nm away from the U6 base. Moreover, as described previously, the C27 base is highly mobile; it is located outside the binding pocket and is completely solvent-exposed in macrostates 1, 6, and 8, yet remains buried within the pocket in macrostates 2, 3, and 5.

As a result of the conformational heterogeneity of the binding site-defining residues, several of the macrostates are characterized by only a partially formed or even a completely missing binding pocket (Fig 8 and S2 Fig). For example, macrostate 3 features a close stacking interaction between G26 and A28, whereas in the NMR structure and the binding-competent unbound structures the G26 and A28 bases are separated by an average distance of ~0.8 nm. This close stacking interaction seals off a large portion of the binding site, which relies on the presence of a significant separation between G26 and A28. Similarly, in macrostates 4 and 7, the bases of A7, C8, C22, G26, and A28 are clustered tightly together, leaving no binding pocket and completely abolishing the ability of theophylline to dock inside the RNA. The average theophylline docking scores to 500 randomly sampled conformations from macrostates 3, 4, and 7 as calculated by AutoDock Vina are 2.8 kcal mol^–1^, 11.0 kcal mol^–1^, and 13.3 kcal mol^–1^, respectively. These positive scores reflect the absence of a binding pocket that can accommodate any of theophylline’s molecular volume (S2 Fig). In macrostate 8 the RNA has only a small, shallow cleft and minimal surface area contact with theophylline, corresponding to an average docking score of –1.3 kcal mol^–1^. However, despite these disruptions to the binding site conformation, macrostates 1, 2, 5, and 6 remain capable of accommodating theophylline and forming stabilizing intermolecular hydrogen bonds.

Overall, the modeled unbound-state conformational landscape of the theophylline aptamer is in qualitative and quantitative agreement with previous kinetic studies showing that only a portion of the RNA population adopts a conformation amenable to theophylline binding. Moreover, the models are consistent with NMR data indicating that the RNA tertiary structure and the theophylline binding site structure, in particular, are not stably formed in the absence of bound theophylline[4,38]. However, the use of MD simulations in conjunction with MSMs offers the advantage of allowing the specific alternative, unstable structures to be visualized at atomic spatial resolution and permitting the dynamical behavior of the RNA to be quantified on the picosecond timescale, including the kinetics of transitions between active and inactive conformations. Additionally, the RNA conformations revealed by our modeling allow us to explain the structural basis for why the binding site in certain macrostates is incapable of accommodating theophylline.

### Comparison of conformational landscapes of theophylline-bound versus unbound RNA

To elucidate the effects of bound theophylline on the RNA conformational landscape relative to the unbound-state landscape, we performed 80 individual 10-ns MD simulations of the theophylline-bound RNA, starting from the NMR structure, yielding a total simulation time of 800 ns. The presence of bound theophylline greatly decreases the range of conformations adapted by the RNA. Throughout the 800 ns of simulation time, the main chain RMSD relative to the starting NMR structure ranges from 0.12 nm to 0.47 nm and has an average value of 0.26 nm. These RMSD values are considerably lower than those for the unbound RNA, which rises as high as 1.4 nm and has an average value of 0.50 nm.

Moreover, the flexibility of most individual residues is lower in the bound RNA, as measured by their main chain root-mean-squared fluctuation (RMSF) values (S3 Fig). Most notably, U32 and C33 are significantly more flexible in the absence of theophylline than they are in the presence of theophylline, having RMSFs that are 0.15 nm greater in the unbound versus in the bound state. Similarly, the majority of binding site residues are more flexible in the unbound state, allowing them to take on the diverse set of conformations shown in Fig 8 and S2 Fig. A notable exception to this overall trend of lower residue flexibility in the bound RNA involves C27, whose RMSF is 0.15 nm greater in the presence of theophylline. In the RNA–theophylline complex, C27 extends outward into the bulk solvent in all analyzed conformations, having a mean base SASA of 1.35 nm^2^, and its base forms no stabilizing interactions with other residues. This lack of stabilizing interactions allows greater flexibility than in the unbound state, when C27 is frequently buried in the binding site (having a mean base SASA of only 0.65 nm^2^) and forms hydrogen bonds with other residues.

The bound RNA also adopts a much smaller diversity of secondary structures compared to the unbound RNA. In contrast to the 39 unique secondary structures observed during the unbound RNA simulations, only 17 unique secondary structures are visited by the bound RNA. The two most frequently occurring secondary structures account for 90% of the analyzed simulation conformation (S4 Fig). These two secondary structures are identical to the NMR structure and identical to one another, with the exception of a missing base pair between G1 and C33 in the most frequent structure. Each of the remaining 15 secondary structures accounts on average for only ~0.7% of conformations.

**Fig 9:**
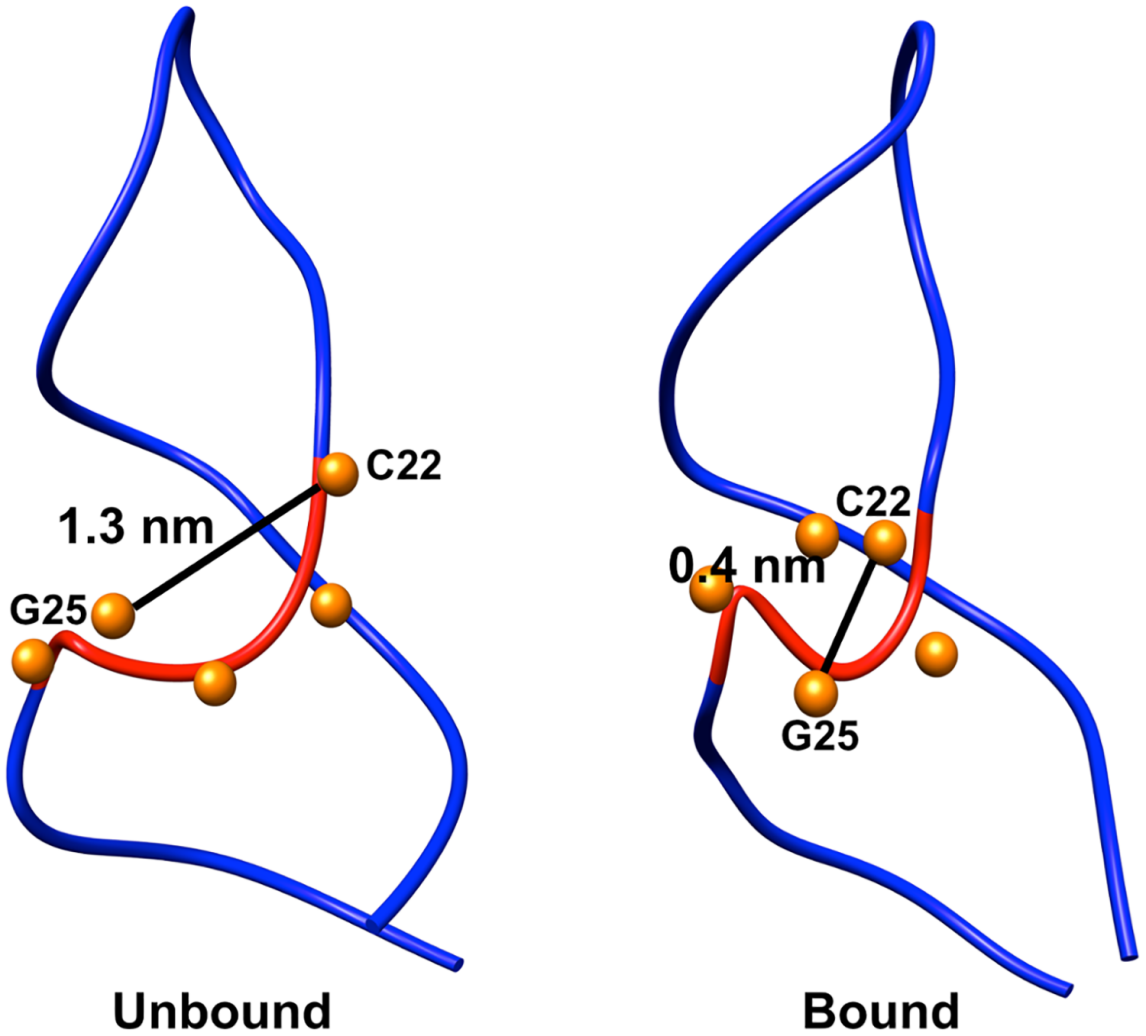
Loss of S-turn between C22 and G26 in unbound RNA. The main chain of residues 22–26 is colored red; phosphorous atoms of residues 22–26 are depicted as orange spheres. Distances between main chain phosphate atoms of C22 and G25 are indicated. The sharp S-turn in the theophylline-bound state is shown for comparison.

Not only does the presence of theophylline stabilize the global structure and binding site structure, but it also stabilizes the S-turn that is formed between C22 and G26. In the bound-state simulations, this sharp S-turn retains a conformation similar to that in the NMR structure and has an average distance of 0.4 nm between the main chain phosphate atoms of C22 and G25. However, when the RNA is unbound the S-turn is greatly distorted, and the average distance between the main chain phosphate atoms of C22 and G25 increases to 1.3 nm (Fig 9). As a result of this decrease in S-turn sharpness, the Mg^2+^ binding site provided by the S-turn is lost in the absence of theophylline. For the simulations in the theophylline-bound state, a Mg^2+^ ion remains positioned at 0.6 nm from the center of mass of the S-turn over the course of all simulations. In contrast, in the theophylline-free simulations the closest Mg^2+^ ion is positioned at an average distance of 1.5 nm. The loss of a Mg^2+^ binding site is presumably the consequence of the greater distance between the backbone phosphates in the S-turn and hence of a reduced driving force for divalent cations to stabilize charge-charge repulsion between these phosphate groups.

### Theophylline binding pathway and binding mechanism

**Fig 10:**
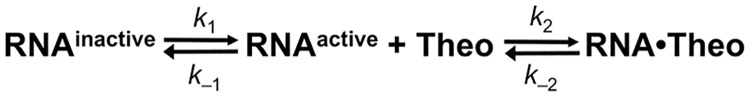
Previously proposed theophylline binding mechanism based on NMR data. (Figure adapted from ref. [4]).

We have combined MD simulations and Markov state modeling to map the complete theophylline binding pathway at a theophylline concentration of 10 mM, starting from theophylline in bulk solution and ending in the fully bound conformation of the NMR structure. Our mapped pathway shows that the theophylline binding mechanism involves conformational selection followed by induced fit. NMR data[4] have demonstrated that only ~33–62% of the population of unbound RNA is in an active, binding-competent conformation (RNA^active^), which is further supported by our modeling in the present study. Consequently, in previous models of theophylline binding the first step in the binding mechanism involves a conformational selection process in which an ensemble of inactive, binding-incompetent RNA conformations (RNA^inactive^) spontaneously undergoes a conformational change to RNA^active^ (Fig 10). Once the conformational change to RNA^active^ has taken place, theophylline associates with the binding site and forms the RNA–theophylline complex.

Kinetic experiments have shown, however, that RNA^active^ is not in an optimal conformation for theophylline binding[4]. The apparent association rate constant *k*_2_ for theophylline binding (~2 x 10^5^ M^–1^ s^–1^) is more than 1000 times slower than that for diffusion-controlled binding[89], whereas for an ideally preformed binding site a value of *k*_2_ near the diffusion limit would be expected. Thus, it has been hypothesized that the slower kinetics of theophylline binding are the result of a process in addition to conformational selection, such as additional conformational rearrangement of the theophylline binding site after ligand association, ligand desolvation, or RNA desolvation[4]. To our knowledge, nevertheless, no studies have resolved which, if any, of these conjectured secondary processes best explains the slow association kinetics. Our modeling here strongly supports the hypothesis that conformational rearrangement of the binding site occurs after the initial association between RNA^active^ and theophylline.

**Fig 11:**
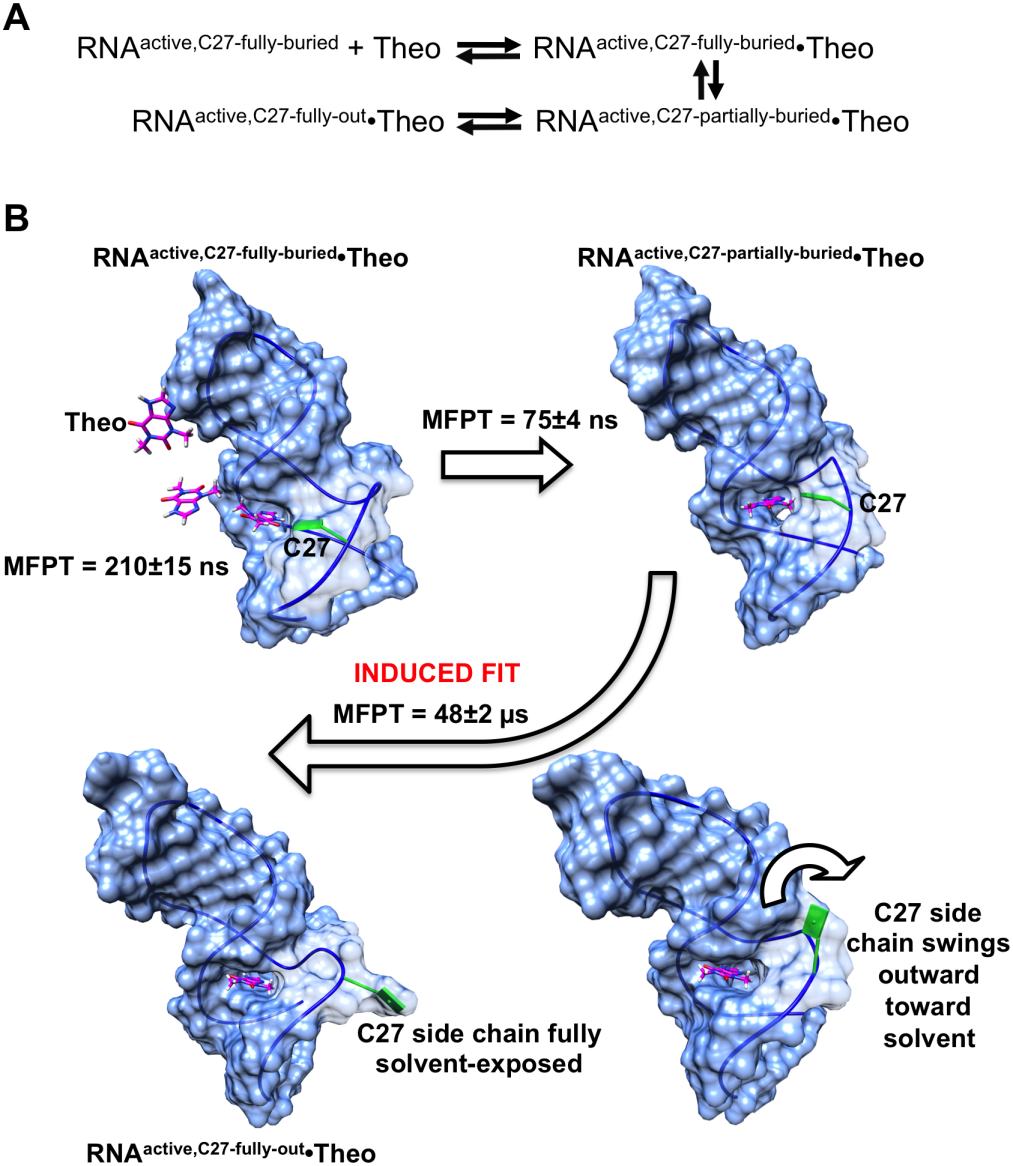
Complete modeled theophylline binding pathway. (A) Schematic showing the main intermediate states between initial diffusion of theophylline into the RNA binding site (RNA^active,C27-fully-buried^ + Theo) and the final, fully associated RNA–theophylline complex observed in the NMR structure (RNA^active,C27-fully-out^•Theo). (B) Molecular view of the binding pathway. Mean first-passage times (MFPT) required to transition between consecutive intermediate states are shown. The conformational transition from the RNA^active,C27-partially-buried^•Theo state, in which theophylline is bound within a non-optimal binding pocket, to the state RNA^active,C27-fully-out^•Theo, in which the binding pocket has reached its final, optimal conformation, is characteristic of an induced fit process. This induced fit follows the previously known conformational selection binding mechanism. Theophylline and the C27 base are colored magenta and green, respectively.

The complete predicted theophylline binding pathway is shown in schematic form in Fig 11A. The centroid structure of the most populated binding-competent unbound macrostate (macrostate 6) was selected as the unbound RNA target conformation for which the pathway is to be modeled. We refer to this unbound starting RNA conformation as RNA^active,C27-fully-buried^ because the RNA is in an active, binding-competent conformation, but the C27 base is completely buried in the binding pocket. It should be noted that since the modeled pathway begins with an RNA^active^ conformation, the conversion from RNA^inactive^ to RNA^active^ is not explicitly considered here; this conversion takes place as part of the continual transitions between macrostates described previously.

The initial phase of the binding pathway, in which theophylline diffuses into the RNA binding site from bulk solution, was modeled first. A MSM with a lag time of 2 ns was constructed for the diffusion process, starting with theophylline in bulk solution 0.3 nm from the binding site. Transition path theory (TPT) was used to compute the flux of the top five pathways between the fully unbound starting state and the end state, in which theophylline has just entered the binding site. The single diffusion pathway with the highest flux (80%) shows theophylline approaching the binding site “backwards,” with its two methyl groups positioned closest to the entrance (Fig 11B). When the center of mass of the ligand is ~0.7 nm from the entrance, the ligand rotates by 180° such that its N7 and N9 atoms point toward the binding site. Theophylline then diffuses partially into the site, with its six-membered ring remaining partly exposed to solvent, forming a weak complex with the RNA. The MFPT for this process of diffusion and initial weak complex formation is 210 ns. We refer to the weak RNA–theophylline complex as RNA^active,C27-fully-buried^•Theo, as the C27 base remains fully buried in the RNA even after initial complex formation. Interestingly, in this weak complex, theophylline has an orientation within the binding site that is inverted relative to its orientation in the final, fully bound complex observed in the NMR structure. In the fully bound complex, atom N7 of theophylline is located proximally to C22 and atom N9 is located proximally to U24 (S5A Fig). However, in the weak complex, N9 is positioned close to C22, while N7 is positioned close to the base of C27 rather than U24; C27 forms an integral part of the binding site in RNA^active,C27-fully-buried^•Theo (S5B Fig). A single non-native hydrogen bond is formed between the N4 atom of C27 and atom O6 of theophylline in RNA^active,C27-fully-buried^•Theo. Despite the formation of this hydrogen bond, RNA^active,C27-fully-buried^•Theo is a weak complex since theophylline is only partially buried and still partly solvent-exposed (S5C Fig). The buried surface area between theophylline and the RNA in the initial weak complex is only 2.6 nm^2^, compared to 3.5 nm^2^ in the fully bound complex.

Approximately 75 ns after theophylline diffuses into the binding site, the C27 base has begun to swing away from its starting position, and theophylline has rotated into its final, "correct" bound orientation, with its N7 and N9 atoms positioned near C22 and U24, respectively (Fig 11B). Moreover, theophylline has entered completely into the binding site, becoming fully buried within the RNA and losing the solvent exposure of its six-membered ring. At this point in the binding process, however, the binding site has not yet reached its final configuration, as the C27 base is still partially buried within the binding site. We refer to this state as RNA^active,C27-partially-buried^•Theo. Thus, the RNA has not yet reached its optimal conformation for theophylline binding.

We constructed an additional MSM with a lag time of 5 ns to map the conformational change from the RNA^active,C27-partially-buried^•Theo state to the final state featuring the optimal binding site conformation of the NMR structure. The latter is designated as RNA^active,C27-fully-out^•Theo. The MSM for this conformational change is based on a set of 300 MD simulations, starting from the RNA^active,C27-partially-buried^•Theo conformation, that were performed with an objective function of minimizing the binding site RMSD between RNA^active,C27-partially-buried^•Theo and the first conformer of the NMR structure. A plot of implied timescales for this MSM is provided in S6 Fig. According to the MSM, after a MFPT of 48 μs, the C27 base has transitioned from its partially buried conformation to its solvent-exposed conformation that characterizes the fully bound state, and the binding site residues have reached their final, optimal conformation (Fig 11B).

These data corroborate the prior hypothesis that a conformational rearrangement after initial complex formation is at least partly responsible for reducing the association rate constant from the diffusion-controlled rate[4]. The modeled requirement for a structural rearrangement connecting RNA^active,C27-partially-buried^•Theo to RNA^active,C27-fully-out^•Theo suggests that theophylline binds to the RNA aptamer through a complex mechanism involving both conformational selection, as previously known, and induced fit. The conformational change of the induced fit process is predicted to occur on a timescale (48 μs) that is ~230 times greater than that of the diffusion of the ligand into the binding site (210 ns). This timescale difference is smaller than the ~1000-fold difference observed in kinetic experiments[4], but the MSM nonetheless indicates the sequence of specific conformational rearrangements that must take place in the binding site in order for the experimentally observed complex to be reached.

### Structural basis for role of Mg^2+^ in enhancing theophylline binding kinetics

Previous studies have shown that Mg^2+^ plays a key role in stabilizing the RNA–theophylline complex, increasing the affinity for theophylline by 10,000-fold relative to that in the absence of Mg^2+^ [4,34,90]. It has also been demonstrated that Mg^2+^ has a major effect on the kinetics of theophylline binding; the apparent *k*_on_ in the presence of 10 mM Mg^2+^ is ~310-fold greater than that in the absence of Mg^2+^ [39]. Furthermore, in the presence of 10 mM Mg^2+^ the lifetime (1/*k*_off_) of the complex is 14 s, while the complex lifetime in the absence of Mg^2+^ is ≤ 50 ms[4]. It is well established that Mg^2+^ is critical for the correct folding and function of many RNAs. As a recent example, Mg^2+^ has been shown to stabilize the folded, ligand binding-competent state of a 49mer RNA ribozyme Diels-Alderase [91]. This general requirement of Mg^2+^ for correct RNA folding and function has led other authors to suggest that for the theophylline aptamer, the presence of Mg^2+^ leads to an increase in the population of binding-competent molecules[39]. Therefore, we sought to assess the structural and dynamical effects of the absence of Mg^2+^ on both the theophylline-bound and theophylline-free states of the RNA at atomic resolution. Total simulation times of 800 ns and 5.4 μs were used to model these two states, respectively, with no Mg^2+^ present.

The absence of Mg^2+^ has little effect on the structure or dynamics of the RNA–theophylline complex. In the absence of Mg^2+^, the global RNA main chain RMSD for the complex relative to the starting NMR structure ranges from 0.21 nm to 0.49 nm and has an average value of 0.33 nm. These RMSD values are comparable to those observed in our simulations of the complex in the presence of 10 mM Mg^2+^, for which the maximum and average main chain RMSD values are respectively 0.47 nm and 0.26 nm. In addition, the RMSD of non-hydrogen atoms of the binding site residues relative to the starting structure ranges from 0.14 nm to 0.27 nm in the absence of Mg^2+^. Collectively, these sets of RMSD values indicate that both the global RNA structure and the binding site structure exhibit roughly the same degree of structural variability regardless of whether 10 mM Mg^2+^ is present. Moreover, the secondary structures of the RNA are identical with and without Mg^2+^ (data not shown). These findings are consistent with experimental studies demonstrating that NMR chemical shifts are very similar for the RNA–theophylline complex in the presence and absence of Mg^2+^, indicating that the structure of the complex does not significantly change with Mg^2+^[4].

**Fig 12:**
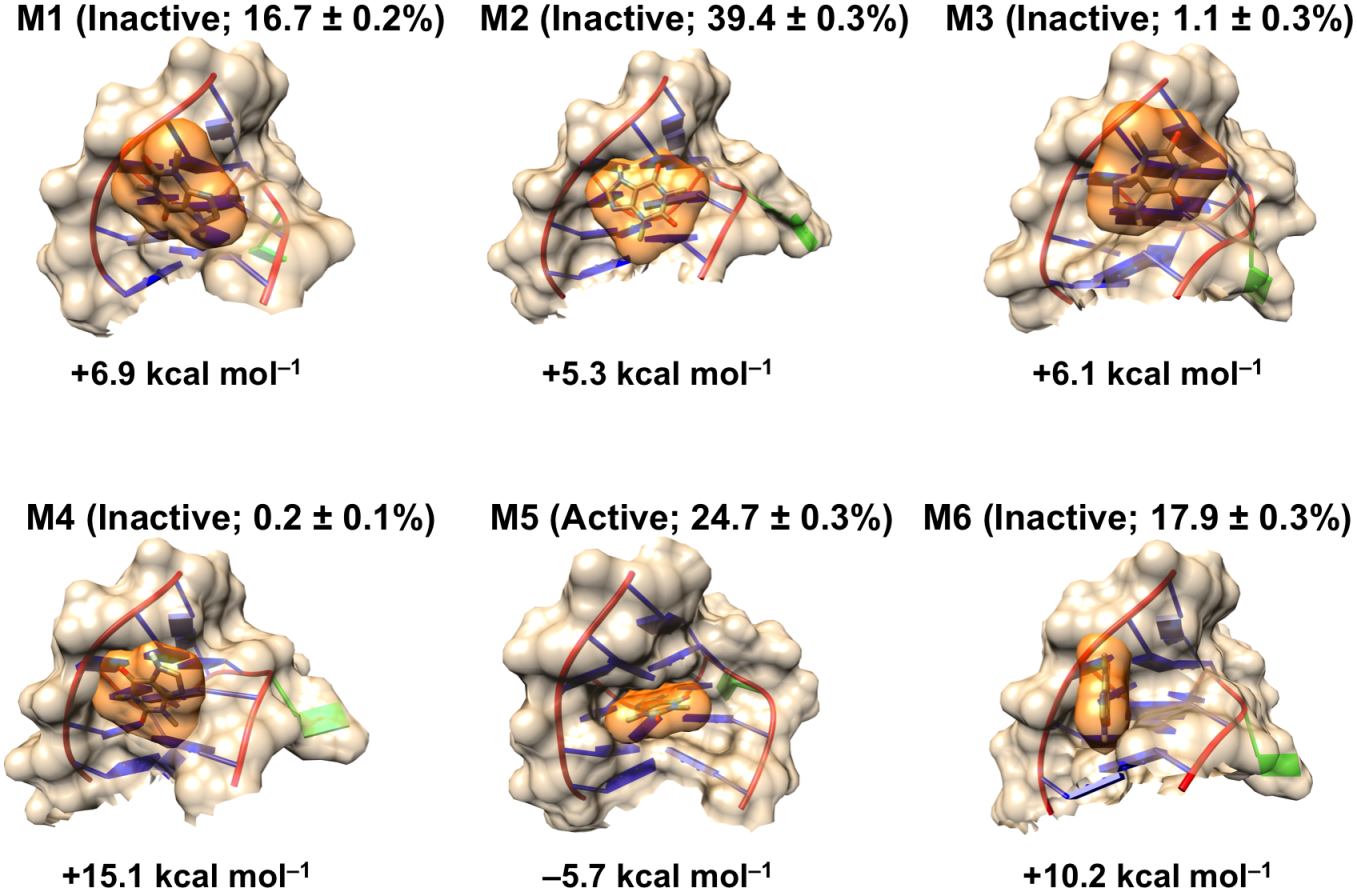
Conformational diversity of unbound RNA in the absence of Mg^2+^. Shown are molecular surfaces for binding site regions (residues 5–9 and 22–29) of centroid structures of the six macrostates (M1-M6) taken from the MSM that was constructed from 5.4 μs of MD simulation data. Binding-competent and binding-incompetent macrostates are labeled as active and inactive, respectively, and relative populations of macrostates are indicated. The average theophylline docking score for each macrostate as calculated by AutoDock Vina is listed below each structure. The best-scoring theophylline pose for each structure is shown as an orange surface.

In marked contrast to the RNA–theophylline complex, when the RNA is unbound, the absence of Mg^2+^ has a profound effect on the conformational landscape. We generated a MSM for the 5.4 μs of simulation time for the unbound RNA without Mg^2+^. The MSM was coarse-grained by BACE to include 6 macrostates in accordance with the plot of the Bayes factor as a function of number of macrostates. The implied timescales for the coarse-grained MSM are shown in S7 Fig. Each macrostate was classified as either binding-competent or binding-incompetent based on the average score generated by docking theophylline to 500 RNA conformations randomly sampled from the macrostate. Macrostates whose average docking score is ≤ –5.0 kcal mol^–1^ are deemed binding-competent, as described previously for the conformational landscape in the presence of Mg^2+^. Strikingly, only macrostate 5, which has a population of ~25% of sampled conformations, is binding-competent (Fig 12). This macrostate has a preformed cavity that accommodates theophylline with good shape complementarity, yielding an average binding score of –5.7 kcal mol^–1^. The other five macrostates, accounting for 75% of the total RNA population, are binding-incompetent and lack binding pockets for theophylline. These populations are in sharp contrast to the unbound RNA in the presence of 10 mM Mg^2+^, where 58% of the population is binding-competent and only 42% is binding-incompetent.

**Fig 13:**
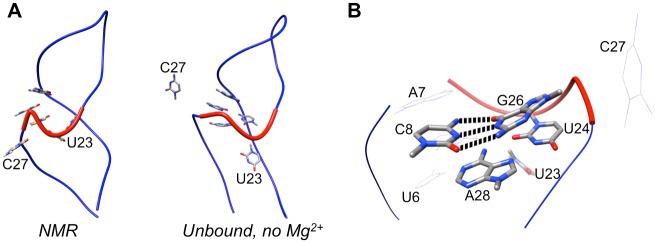
Absence of Mg^2+^ distorts the S-turn formed by residues 22–26 and prevents binding site formation. (A) Ribbon diagrams of the NMR structure (left) and global centroid structure of the five binding-incompetent macrostates from the MD simulations of unbound RNA in the absence of Mg^2+^. The S-turn region is shown as a wider ribbon colored red. (B) Binding site residues of the global centroid structure. A28 stacks with the C8:G26 base pair, forming a cluster of three residues that prevents binding pocket formation. Ribbon coloring and S-turn region depiction are the same as in panel A.

Our modeling reveals a surprising effect of the absence of Mg^2+^ whereby the C27 base becomes completely exposed to solvent, even without bound theophylline. The average SASA of the C27 base across 5.4 μs of simulation time is 1.0 ± 0.35 nm^2^ (mean ± standard deviation), which is much greater than the average SASA of 0.65 ± 0.20 nm^2^ for the unbound aptamer in the presence of 10 mM Mg^2+^, where C27 is mostly buried. This solvent-exposure of C27 results from perturbation of the S-turn formed by residues 22–26. Since no Mg^2+^ ions are present to shield phosphate-phosphate repulsions, the S-turn is severely distorted in the five binding-incompetent macrostates, becoming greatly lengthened and losing almost all of its S-like character (Fig 13). Consequently, U23 moves out of the binding site, becoming partially solvent-exposed. U24 no longer stacks with A28, but instead becomes coplanar with A28 and C22, forming a base triple. Having lost its stacking with U24, A28 instead stacks with G26 and C8, which in turn form a base pair with one another. The new stacking interaction between A28 and the C8:G26 base pair prevents cavity formation.

Additionally, we find that Mg^2+^ influences the unbinding kinetics of theophylline not through direct interactions between the Mg^2+^ ion and theophylline, but primarily through the effect of Mg^2+^ on the RNA structure. In order to differentiate between these two possibilities, we predicted the lifetime (1/*k*_off_) of the RNA–theophylline complex in three scenarios: (i) natively folded RNA in the presence of 10 mM Mg^2+^; (ii) natively folded RNA in the absence of Mg^2+^; and (iii) non-natively folded RNA in the presence of 10 mM Mg^2+^. Here, we use the term ‘natively folded’ to refer to the first RNA conformation of the NMR structure. We use the term ‘non-natively folded’ to refer to RNA conformations corresponding to the unbound aptamer in the absence of Mg^2+^. If direct interactions between Mg^2+^ and theophylline are mainly responsible for altering unbinding kinetics, then we would expect that the lifetime of the complex in scenarios (i) and (ii) would be substantially different, as these scenarios differ not in terms of RNA structure but only by the presence of Mg^2+^. However, if the effect of Mg^2+^ on RNA structure (via stabilization of the binding site) is mainly responsible for altering kinetics, then we would expect to see a substantial difference in complex lifetimes between scenarios (i) and (iii), which differ only in terms of RNA structure. Using well-tempered metadynamics, we accelerated the unbinding of theophylline from the NMR structure for scenarios (i) and (ii) and from the global centroid structure of the 5.4 μs of MD simulations of the unbound aptamer without Mg^2+^. The unbinding simulations for scenarios (i) and (iii) both used 10 mM Mg^2+^, while those for scenario (ii) lacked Mg^2+^. The lifetimes of the RNA–theophylline complex for scenarios (i)-(iii) were predicted respectively as 54 ± 43 s, 45 ± 39 s, and 750 ± 730 ms (mean ± standard error). Standard errors were estimated by 10,000 rounds of bootstrapping. The estimated lifetimes for scenarios (i) and (ii) are similar and agree reasonably well with the experimentally measured lifetime of 14 s when [Mg^2+^] = 10 mM[4]. In contrast, the estimated lifetimes for the complex in scenarios (i) and (iii) differ by almost two orders of magnitude, which is slightly smaller than the experimentally measured difference between lifetimes in the presence and absence of Mg^2+^ (14 s and ≤ 50 ms). Given these predicted lifetimes, our results support the hypothesis that the difference in unbinding kinetics caused by Mg^2+^ stems mainly from Mg^2+^-mediated differences in RNA structure rather than from direct interaction between the ion and theophylline. Although we studied only unbinding kinetics, it is probable that the difference in binding kinetics (*k*_on_) associated with Mg^2+^ is also attributable to differences in RNA structure induced by the presence of Mg^2+^.

Taken together, these findings support the hypothesis that Mg^2+^ does not change the true kinetics of theophylline binding, but simply leads to an increase in the population of binding-competent molecules[39]. According to our MSMs, the presence of Mg^2+^ increases the population of binding-competent states more than twofold relative to Mg^2+^-free conditions (58% versus 25%). This effect appears to be mediated largely by the S-turn of residues 22–26. When Mg^2+^ is lacking, there is little driving force for the S-turn to form, as there are no divalent ions to shield repulsion between main chain phosphate groups that would be positioned close to one another if the S-turn were present. As a result of the missing S-turn, in ~75% of the RNA population there is a drastic rearrangement of the nucleotides that normally constitute the theophylline binding site. A double layer of nucleotides comprising A28 and a C8:G26 base pair impedes cavity formation, preventing theophylline binding.

## Conclusion

In this work, we have combined ~21 μs of MD simulations and MSMs to probe the conformational landscape and structural diversity of a model theophylline RNA aptamer in its ligand-free state. This combined modeling approach has allowed us to probe at atomic resolution an unbound aptamer’s structure, which has remained difficult to accomplish by experiment. Our modeling suggests that the theophylline aptamer’s unbound-state landscape features eight principal metastable states, and that transitions between the metastable states occur on the nanosecond to microsecond timescale. The tertiary structures of the aptamer vary greatly among the metastable states, with the greatest conformational variation occurring in the GAAA tetraloop and in the theophylline binding site region. We find that the binding site nucleotides, in particular, explore a great diversity of conformations, about 40% of which either completely abolish the binding pocket or shrink it to such an extent that only partial burial of theophylline and weak binding take place. These binding-incompetent states must undergo conformational changes to binding-competent states with preformed binding pockets in order for theophylline to form an RNA–theophylline complex. This finding agrees with experiment but provides greater structural detail than what NMR studies have so far been able to provide. Moreover, our modeled theophylline binding pathway indicates that a notable conformational rearrangement of the RNA binding site occurring on the ~50-μs timescale is required after theophylline diffuses into the binding site, accompanied by C27 transitioning from a buried to completely solvent-exposed orientation. This conformational change appears to be a major contributor to the discrepancy seen experimentally between the actual theophylline association rate constant and the diffusion-controlled rate constant and suggests that theophylline binding occurs via a combination of conformational selection and induced fit mechanisms. Finally, simulating the RNA in the absence of Mg^2+^ indicates that loss of Mg^2+^ causes the population of binding-competent RNA molecules to decrease more than twofold relative to a 10 mM Mg^2+^ concentration. The modeled theophylline unbinding kinetics in the presence and absence of Mg^2+^ support prior conjecture that the drastic effects of Mg^2+^ on *k*_off_ and, by extension, on *k*_on_ are mediated not by direct interactions between the ion and theophylline, but by structural destabilization of the S-turn and binding site that occur when Mg^2+^ is not present.

From a technical perspective, the computational method presented here opens up the possibility to analyze the unbound structures and dynamics of other engineered and naturally occurring RNA aptamers at atomic and picosecond resolution, as well as to map their ligand binding pathways. To this end, there are several remaining questions that could be addressed. For example, to what extent do other engineered aptamers also exist as ensembles of binding-incompetent and binding-competent states that must interconvert from the former to the latter prior to ligand binding, and what are the specific structural changes that occur? Additionally, do other engineered aptamers employ a combination of conformational selection and induced fit in their ligand binding mechanisms, or is an exclusive conformational selection or induced fit mechanism most prevalent? Answering these and related questions by modeling coupled with experiment could allow a better overall understanding of aptamer function and binding mechanisms. This, in turn, could lead to enhanced aptamer engineering and manipulation of aptamer binding properties for therapeutic and sensor applications.

## Supporting information

S1 Fig Chapman-Kolmogorov test for macrostate and microstate Markov state models of unbound RNA. The Chapman-Kolmogorov test is depicted for (A) each of the states M1-M8 of the 8-macrostate MSM and for (B) microstates 100, 1000, 2000 and 5000 of the 5000-microstate MSM. Values of *p*MSM(A, A; *kτ*) (hollow dots) and *p*MD(A, A; *kτ*) (solid dots) are shown. Error bars represent uncertainties in values of *p*MD(A, A; *kτ*).
(TIF)

S2 Fig A binding pocket large enough to accommodate theophylline is absent in several macrostates of the unbound RNA. The lowest-energy (top-scoring) docked theophylline pose for each RNA macrostate centroid structure is depicted. Listed below the RNA images are average AutoDock Vina scores for theophylline docking to 500 randomly sampled conformations from each macrostate. Molecular surfaces of theophylline are colored orange, and the bases of the RNA residues that define the theophylline binding site in the bound state are colored blue. The base of nucleotide C27 is shown in green. The NMR structure is shown at bottom right. Macrostate populations and active/inactive designations are indicated in parentheses.
(TIF)

S3 Fig RNA residue flexibility differences between theophylline-unbound and theophylline-bound states. Per-residue root-mean-squared fluctuation (RMSF) differences are depicted. Darker red corresponds to a greater RMSF in the unbound state relative to in the bound state. Darker blue corresponds to greater RMSF in the bound state relative to the unbound state.
(TIF)

S4 Fig Two most frequently observed secondary structures of the RNA in the theophylline-bound state. Numbers denote proportions of all analyzed simulation snapshots in which the RNA adopts the depicted secondary structures. Bases that participate in canonical base pairing interactions are depicted in light blue circles. Unpaired bases are depicted in light yellow circles. The secondary structure of the bound-state NMR structure is shown on the right. Secondary structure diagrams were generated by the RNApdbee webserver[88].
(TIF)

S5 Fig Comparison of theophylline orientation in initial weak complex (RNA^active,C27-fully-buried^•Theo) and in fully bound complex. (A) In the fully bound complex/NMR structure, N7 and N9 of theophylline are located close to the bases of C22 and U24, respectively. Both N7 and N9 participate in hydrogen bonding interactions with the respective RNA bases. (B) In the initial weak complex, N7 and N9 of theophylline are positioned near the bases of C27 and C22, respectively. A non-native hydrogen bond occurs between C27 and O6 of theophylline. (C) In the initial weak complex, the six-membered ring of theophylline protrudes outward from the binding pocket, leaving the ring partly exposed to solvent.
(TIF)

S6 Fig Implied timescales of coarse-grained Markov state model of induced-fit binding process. (TIF)

S7 Fig Implied timescales of coarse-grained Markov state model of unbound RNA in the absence of Mg^2+^. (TIF)
