# Supplementary figures and images for: Molecular simulations and Markov state modeling reveal the structural diversity and dynamics of a theophylline-binding RNA aptamer in its unbound state
Source: PLoS One. 2017 Apr 24;12(4):e0176229. doi: 10.1371/journal.pone.0176229 (PMC5402969; doi:10.1371/journal.pone.0176229)

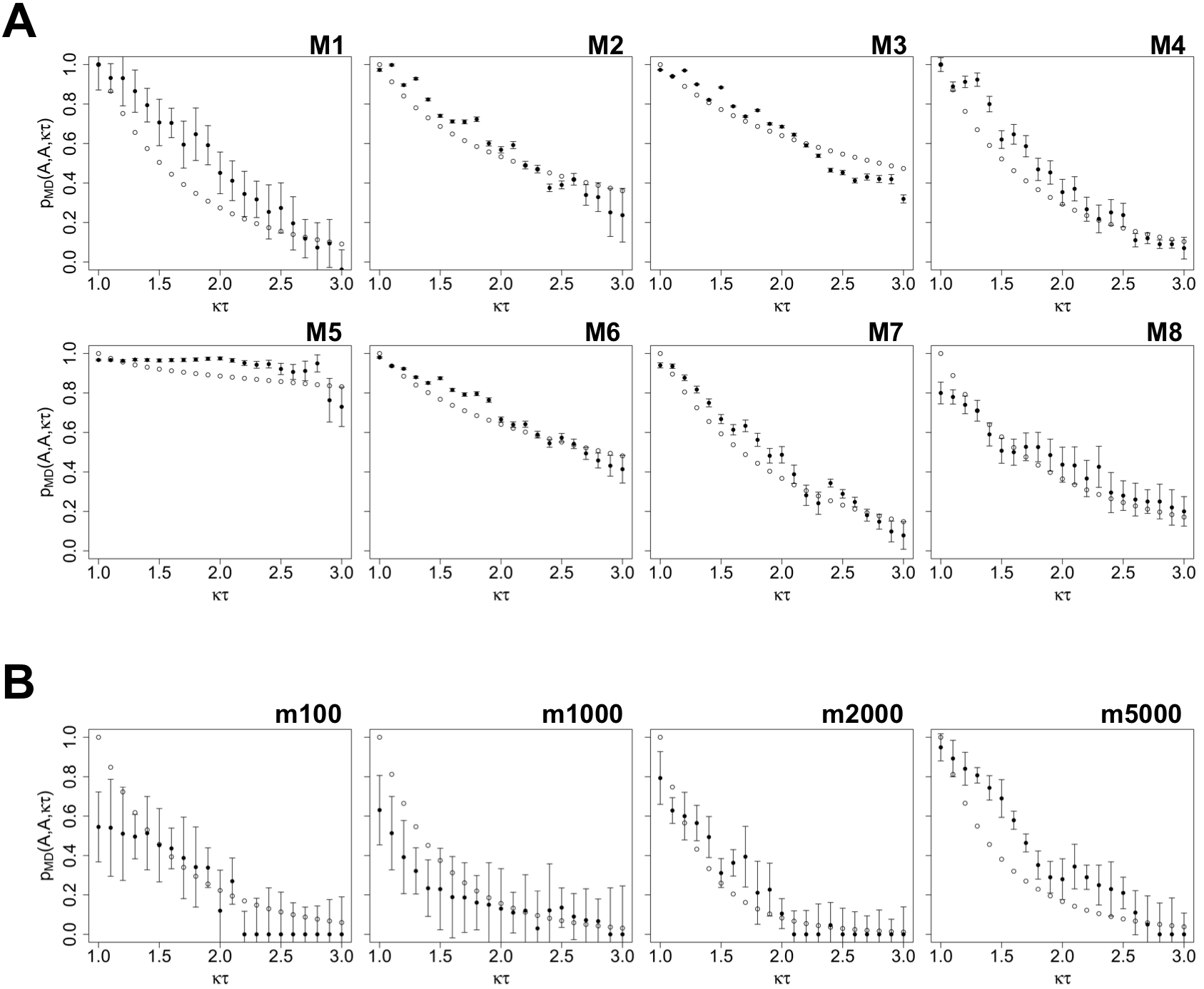

Supplement: S1 Fig — The Chapman-Kolmogorov test is depicted for (A) each of the states M1-M8 of the 8-macrostate MSM and for (B) microstates 100, 1000, 2000 and 5000 of the 5000-microstate MSM. Values of pMSM(A, A; kτ) (hollow dots) and pMD(A, A; kτ) (solid dots) are shown. Error bars represent uncertainties in values of pMD(A, A; kτ). (TIF) [file pone.0176229.s001.tif]

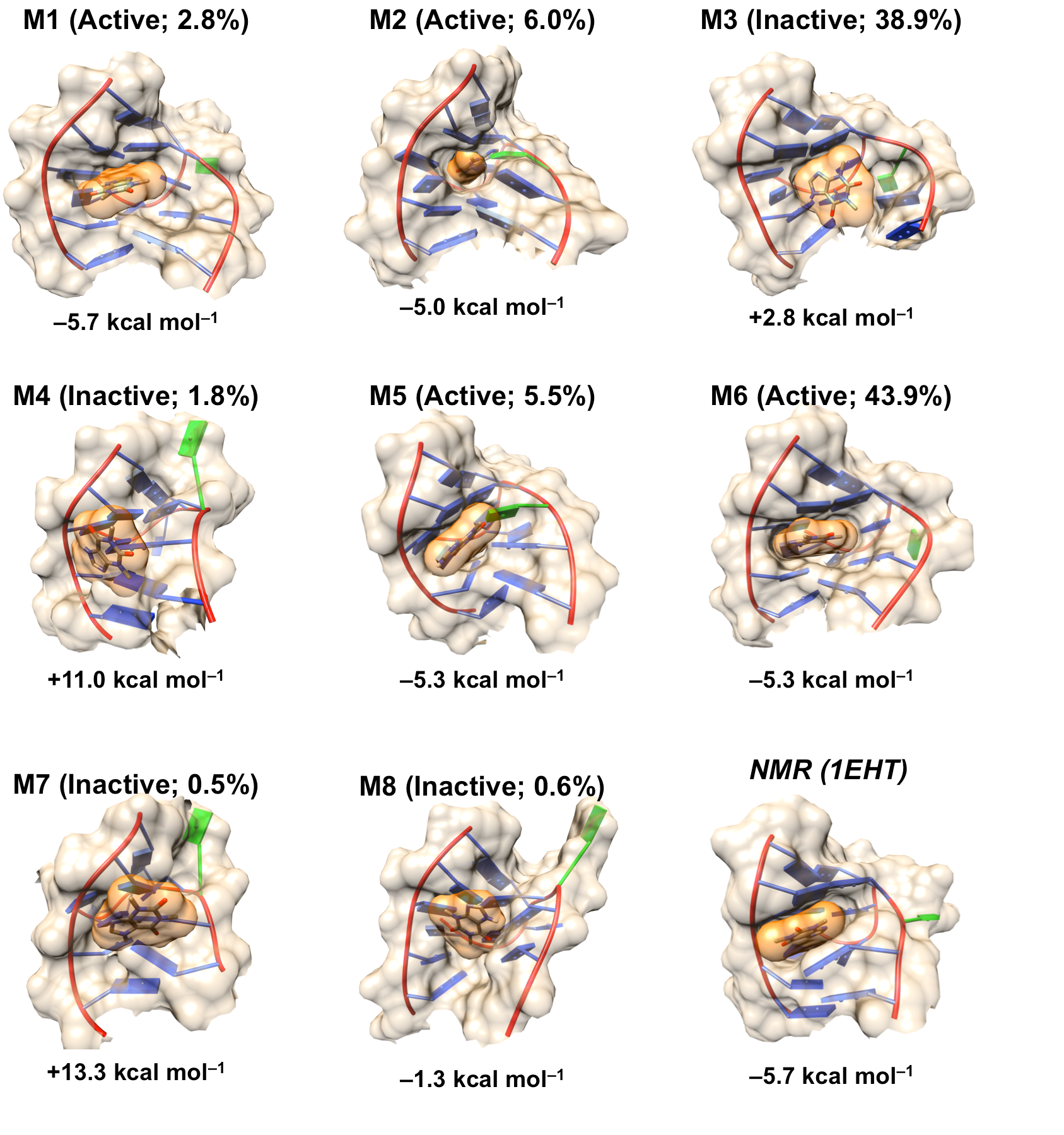

Supplement: S2 Fig — The lowest-energy (top-scoring) docked theophylline pose for each RNA macrostate centroid structure is depicted. Listed below the RNA images are average AutoDock Vina scores for theophylline docking to 500 randomly sampled conformations from each macrostate. Molecular surfaces of theophylline are colored orange, and the bases of the RNA residues that define the theophylline binding site in the bound state are colored blue. The base of nucleotide C27 is shown in green. The NMR structure is shown at bottom right. Macrostate populations and active/inactive designations are indicated in parentheses. (TIF) [file pone.0176229.s002.tif]

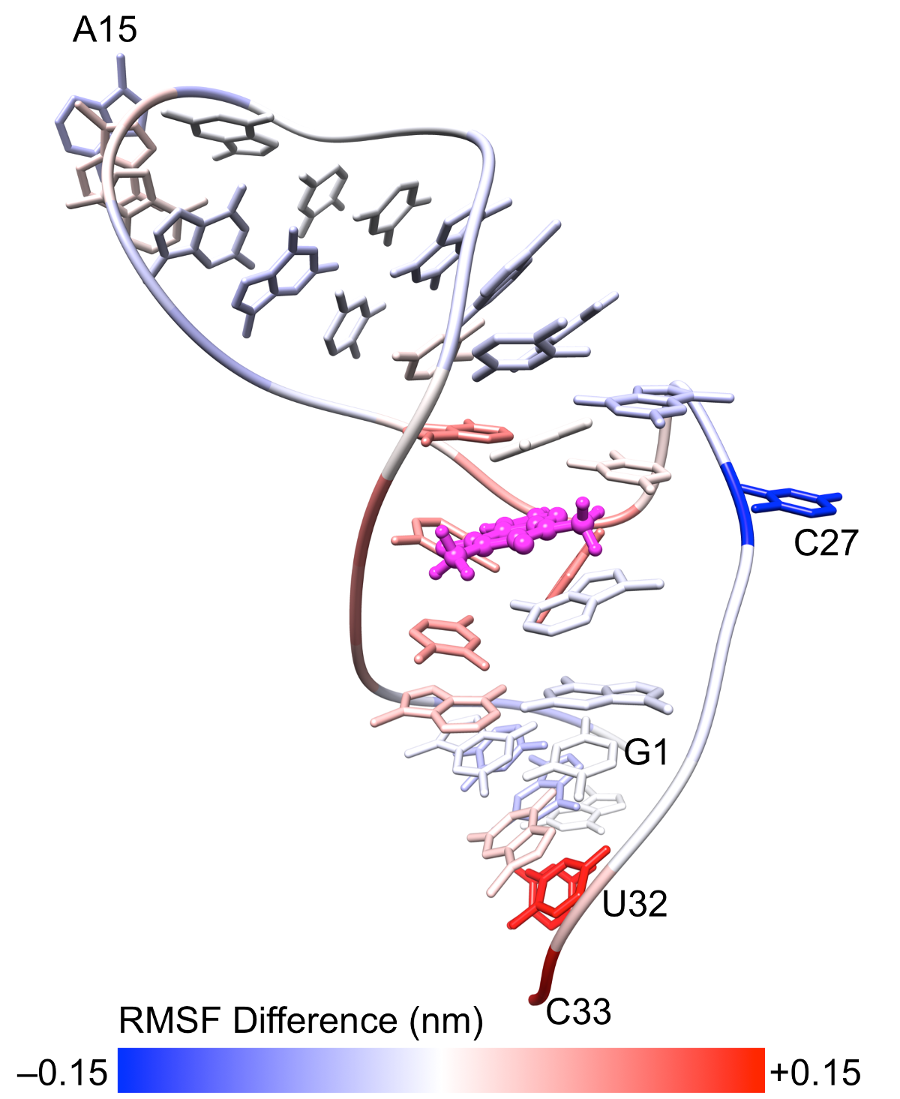

Supplement: S3 Fig — Per-residue root-mean-squared fluctuation (RMSF) differences are depicted. Darker red corresponds to a greater RMSF in the unbound state relative to in the bound state. Darker blue corresponds to greater RMSF in the bound state relative to the unbound state. (TIF) [file pone.0176229.s003.tif]

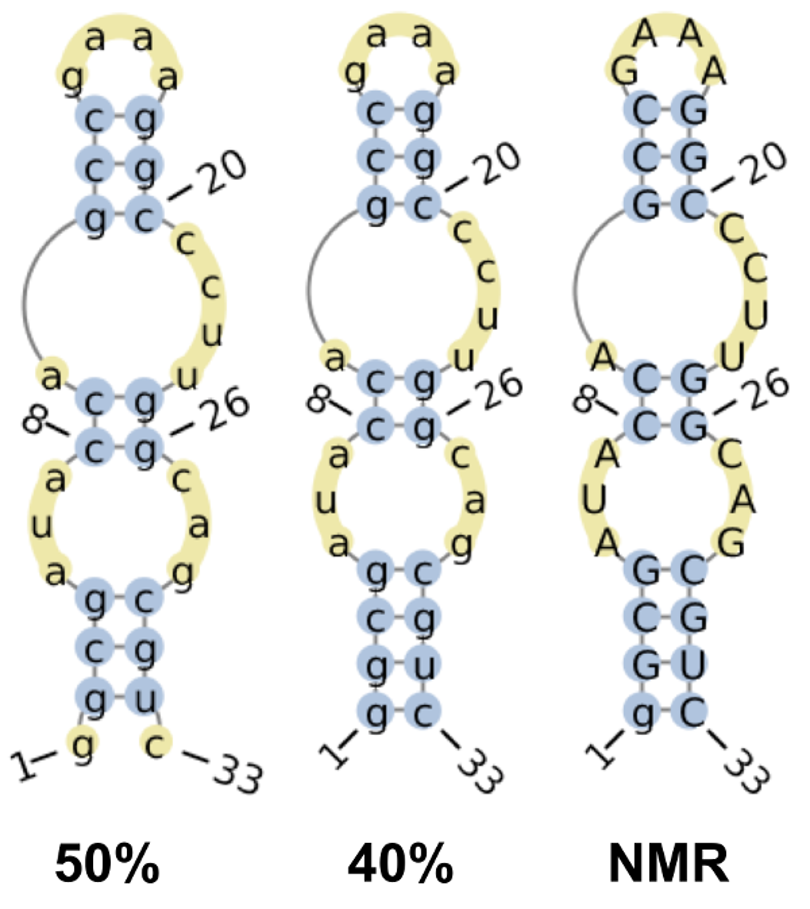

Supplement: S4 Fig — Numbers denote proportions of all analyzed simulation snapshots in which the RNA adopts the depicted secondary structures. Bases that participate in canonical base pairing interactions are depicted in light blue circles. Unpaired bases are depicted in light yellow circles. The secondary structure of the bound-state NMR structure is shown on the right. Secondary structure diagrams were generated by the RNApdbee webserver[88]. (TIF) [file pone.0176229.s004.tif]

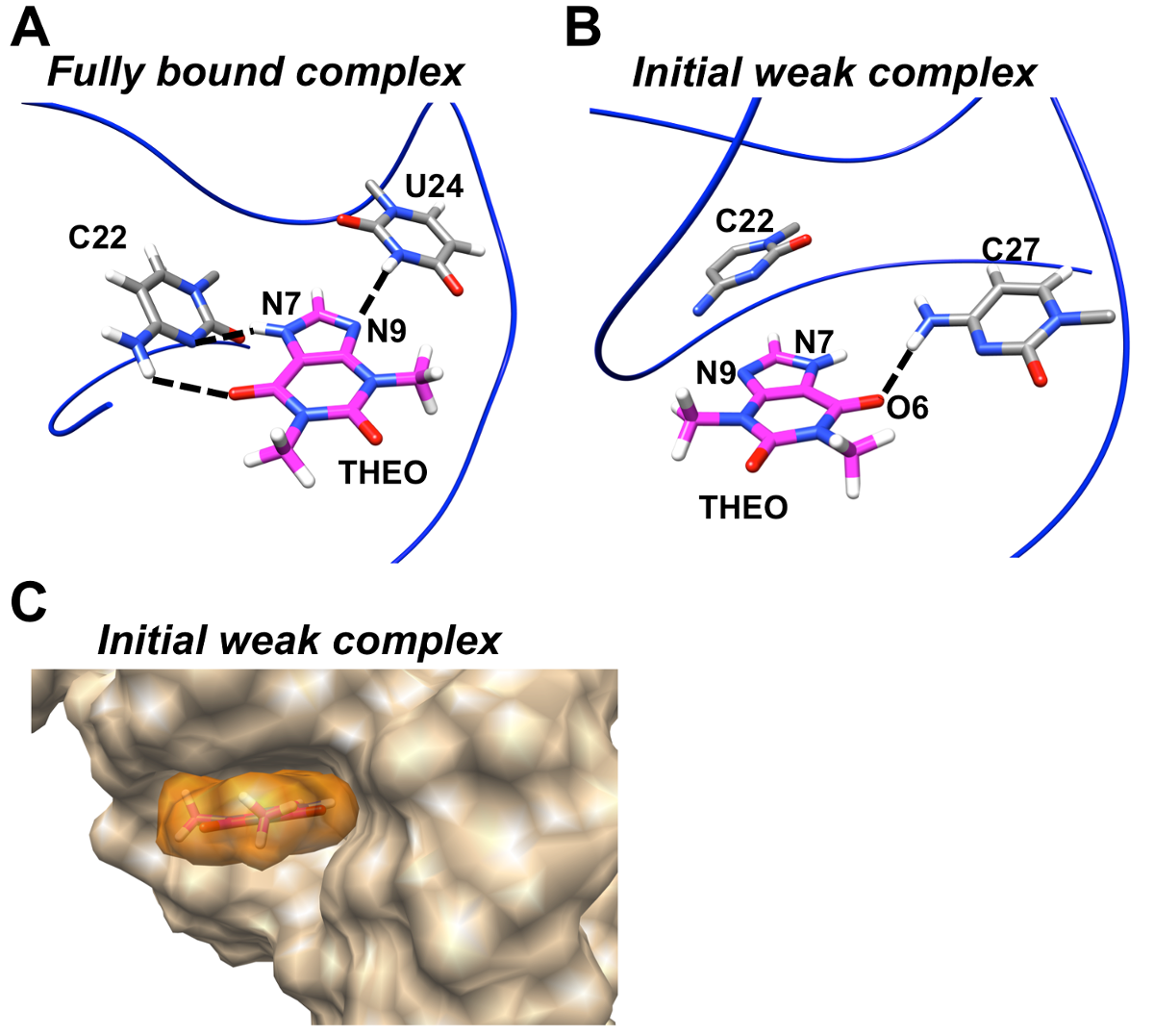

Supplement: S5 Fig — (A) In the fully bound complex/NMR structure, N7 and N9 of theophylline are located close to the bases of C22 and U24, respectively. Both N7 and N9 participate in hydrogen bonding interactions with the respective RNA bases. (B) In the initial weak complex, N7 and N9 of theophylline are positioned near the bases of C27 and C22, respectively. A non-native hydrogen bond occurs between C27 and O6 of theophylline. (C) In the initial weak complex, the six-membered ring of theophylline protrudes outward from the binding pocket, leaving the ring partly exposed to solvent. (TIF) [file pone.0176229.s005.tif]

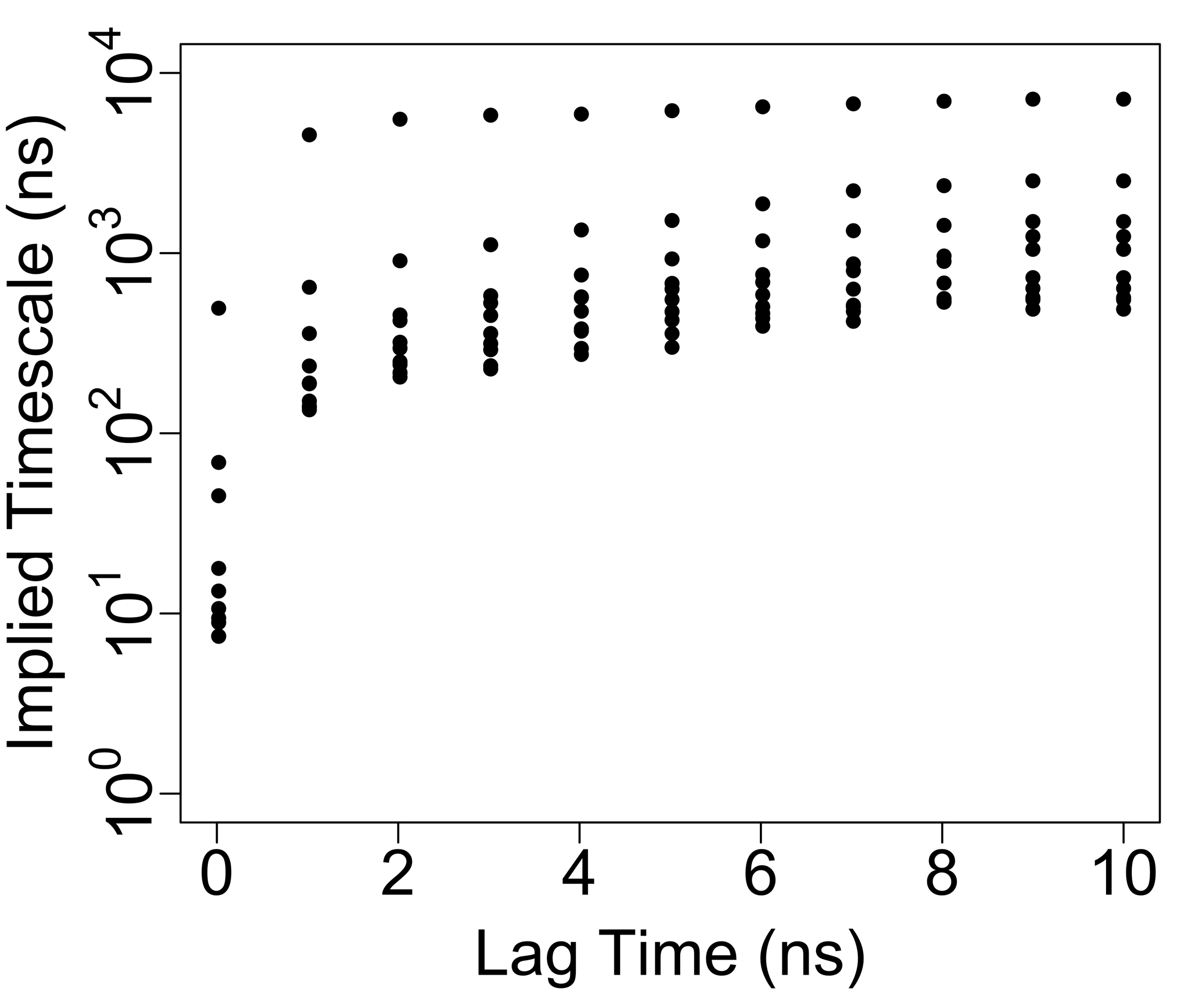

Supplement: S6 Fig — (TIF) [file pone.0176229.s006.tif]

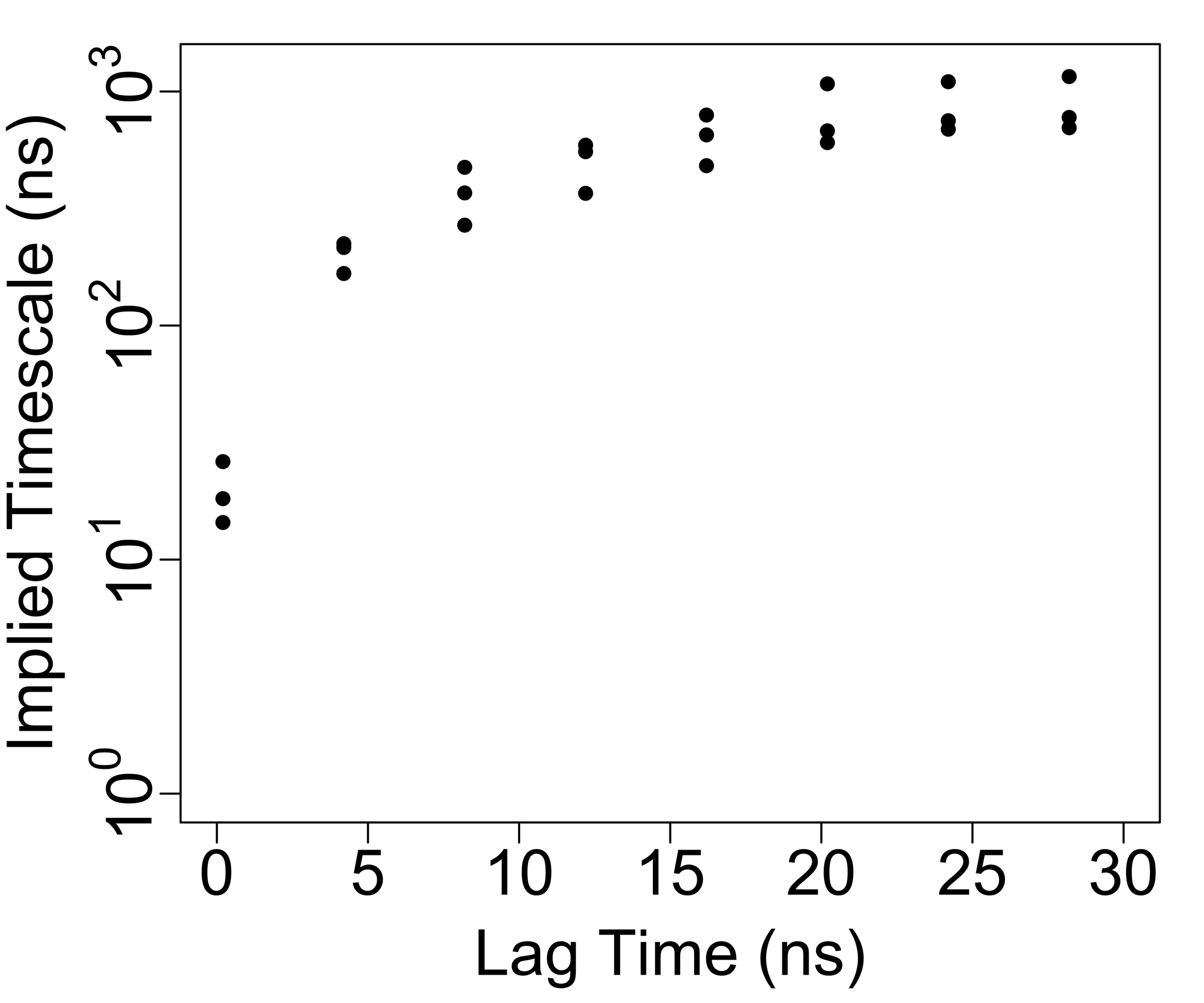

Supplement: S7 Fig — (TIF) [file pone.0176229.s007.tif]
